# Prevalent low Mediterranean diet adherence and low folate status in a Spanish Km 0 Mediterranean coast population

**DOI:** 10.1016/j.crfs.2025.101217

**Published:** 2025-10-07

**Authors:** Ailende Eigbefoh-Addeh, Albert Salas-Huetos, Carla Ramos-Rodríguez, Santiago Ceruelo, Lídia Ríos, Per M. Ueland, Klaus Meyer, Joan D. Fernandez-Ballart, Michelle M. Murphy

**Affiliations:** a Unit of Preventive Medicine and Biostatistics, Faculty of Medicine and Health Sciences, Universitat Rovira I Virgili, IISPV, ANUT-DSM Research Group, 43201, Reus, Spain; b CIBEROBN, ISCIII, 28029, Madrid, Spain; c Department of Nutrition, Harvard T.H. Chan School of Public Health, Harvard University, US-02115, Boston, MA, USA; d Institut d’Investigació Sanitària Pere Virgili (IISPV), 43204, Reus, Spain; e Centre d’Atenció Primària, El Morell, Tarragona, 43760, Spain; f Hospital Lleuger Antoni de Gimbernat de Cambrils, Cambrils, Tarragona, 43850, Spain; g Bevital AS, Bergen, Norway

**Keywords:** B-vitamins, Biomarkers, Dietary intake, Mediterranean diet adherence, Vitamin deficiency

## Abstract

**Background:**

Folate, vitamin B_12_, riboflavin, and vitamin B_6_, play interrelated roles in one-carbon metabolism, vital for several physiological processes.

**Objective:**

To assess intake and status of these B-vitamins in an adult Mediterranean population unexposed to mandatory food fortification or B-vitamin supplementation, and to examine the associations with Mediterranean diet adherence (MDA).

**Methods:**

A representative sample of adults aged 18–77 (n = 740) provided fasting blood samples and 3-day dietary records. MDA was assessed using the Trichopoulou score. Plasma folate, red blood cell folate (RBCF) and plasma vitamin B_12_ were measured via microbiological assays. Riboflavin and vitamin B_6_ status were determined by erythrocyte glutathionine reductase (EGRAC) and aspartate aminotransferase activation coefficients (EASTAC), respectively.

**Results:**

Intake below average requirement was observed in 39.5 % (for folate), 6.5 % (for vitamin B_12_), 20.1 % (for riboflavin), and 19.6 % (for vitamin B_6_) of the participants. Plasma folate <7 nmol/L, plasma B_12_ < 148 pmol/L, RBCF <340 nmol/L, EGRAC ≥1.4, and EASTAC ≥1.6 occurred in 18.6 %, 1.6 %, 1.5 %, 35.9 %, and 56.0 % of participants, respectively. Overall, 82.64 % of participants had low MDA (score <6). Participants in the highest MDA quartile had higher folate intake (354.5 vs. 214.9 μg/day; *P* < 0.001), plasma folate (14.5 vs. 10.0 nmol/L; *P* < 0.001), and RBCF (895.7 vs. 692.8 nmol/L; *P* < 0.001) compared to those in the lowest quartile (score ≤2). Nevertheless, 15.5 % of high-MDA individuals did not meet the folate average requirements, and 8.5 % were folate deficient.

**Conclusions:**

High vs low MDA was associated with better folate intake and status but this was not true for the other vitamins. Only 17.4 % of the population had high MDA and folate deficiency still occurred in this group. A fortification policy may be required, to prevent folate deficiency in the population.

## Highlights

• Only 17.4 % of a Mediterranean population had good Mediterranean diet adherence.
• B-vitamins deficiencies occurred even with good Mediterranean diet adherence.
• 74.1 % of young women had suboptimal folate status to prevent neural tube defects.
• Older participants had more adherence to the Mediterranean diet than younger ones.
• Adequate folate intake does not ensure adequate folate status.

## Graphical abstract

**Figure:**
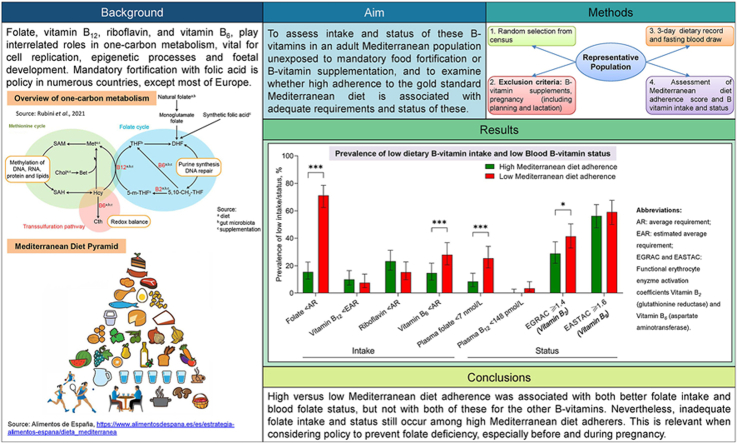

## 1 Introduction

The B-vitamins, folate (B_9_), cobalamin (B_12_), riboflavin (B_2_), and pyridoxine (B_6_) play key and inter-dependent roles as substrates, coenzymes and cofactors in the one carbon metabolic network that regulates DNA synthesis and repair, protein synthesis, and methyl group generation/cycling and donation to methylation reactions (Depeint et al., 2006; Locasale, 2013; Fox and Stover, 2008). Single nucleotide polymorphisms affecting the functions of these vitamins include *methylenetetrahydrofolate reductase* (*MTHFR*) 677 C > T (rs1801133) (Frosst et al., 1995), *reduced folate carrier* [solute carrier family 19A, member 1 (*SLC19A1*)] 80 G > A (rs1051266) (Chango et al., 2000), *methionine transferase reductase* (*MTRR*) 66 A > G (rs1801394) (Wilson et al., 1999), *methionine transferase reductase* (*MTRR*) 524 C > T (rs1532268) (Olteanu et al., 2002), *methionine transferase* (*MTR*) 2756 A > G (rs1805087) (Leclerc, 1996), *transcobalamin II* (*TCII*) 776 C > G (rs1801198) (Namour et al., 2001) and *transcobalamin II* (*TCII*) 67 A > G (rs9606756) (Afman et al., 2002).

The homozygote variant genotype of the *MTHFR* 677 C > T polymorphism (Frosst et al., 1995) has been associated with lower folate status and increased fasting plasma total homocysteine concentration (tHcy) compared to the normal homozygote genotype (Bueno et al., 2016; Jacques et al., 1996) and had the greatest influence on folate status out of various polymorphisms affecting folate metabolism (Bueno et al., 2016). The polymorphism leads to the partial dissociation of the enzyme's FAD cofactor (Frosst et al., 1995), leading to loss of enzyme activity and reduced conversion of 5,10-methylenetetrahydrofolate to 5-methytetrahydrofolate (Yamada et al., 2001). The association between the polymorphism and tHcy has been reported to be greatly attenuated in the presence of optimal riboflavin status (García-Minguillán et al., 2014). The probability of low folate status and elevated Hcy is further enhanced in the combined double homozygote variant genotype resulting from the *MTHFR* 677 C > T and *SLC19A1* 80 G > A (Chango et al., 2000) polymorphisms (Bueno et al., 2016). Folate and vitamin B_12_ play interdependent roles in the remethylation of homocysteine to methionine via methionine synthase. Low dietary supply of vitamin B_12_ due to vegetarian or vegan diets impairs this process (Depeint et al., 2006; Fox and Stover, 2008). Methionine transferase reductase (MTRR), a flavoprotein, maintains MS activity through the conversion of cob(II)alamin to methylcob(III)alamin (Leclerc et al., 1998; Olteanu and Banerjee, 2001). This is also the case in the presence of the *MTRR* 66 A > G and *MTRR* 524 C > T polymorphisms that have been associated with reduced affinity of MTRR leading to less efficient methionine synthase reactivation (Wilson et al., 1999; Olteanu et al., 2002). Riboflavin status also interacts in the association between the *MTRR* 66 A > G polymorphism and Hcy (García-Minguillán et al., 2014). The *MTR* 2756 A > G polymorphism has also been associated with reduced enzyme activity and elevated homocysteine levels (Harmon et al., 1999). Previous studies suggest that the *TCII* 776 C > G (Namour et al., 2001) polymorphism may reduce the binding affinity of transcobalamin and its ability to transport vitamin B_12_ into tissues, potentially reducing intracellular bioavailability and impairing homocysteine metabolism (Namour et al., 2001; Aléssio et al., 2007; Von Castel-Dunwoody et al., 2005). Nevertheless, *TCII* 67 A > G (Afman et al., 2002) genotypes have not been associated with variations in serum holotranscobalamin (holoTC) or plasma B_12_ concentrations (Afman et al., 2002; Fredriksen et al., 2007).

Given these nutrient-nutrient and nutrient-gene interactions, public health measures should consider the requirements of all of the vitamins involved rather than targeting only one vitamin (Titcomb and Tanumihardjo, 2019). B-vitamin status imbalance due to inadequate intake, utilization or excess of any of the vitamins can lead to increased risk of various cancers, polyneuritis, anaemia, neuropathy, renal failure, epileptic seizures, dermatitis and neural tube defects (NTDs) (Hanna et al., 2022). Furthermore, individuals adhering to only plant-based diets without adequate supplementation are at high risk of vitamin B_12_ deficiency (Smith et al., 2018; Green et al., 2017).

Unlike more than 80 countries, mandatory food fortification with vitamins and minerals has not been implemented in most European countries (De Lourdes Samaniego-Vaesken et al., 2012). Many countries, including Spain, permit the sale of voluntary fortified foods with B-vitamins and other micronutrients (Samaniego-Vaesken et al., 2016). The Mediterranean diet is renowned for its ability to support overall nutritional health (Willett et al., 1995; Guasch‐Ferré and Willett, 2021). We hypothesized that the Mediterranean diet provides adequate folate, vitamin B_12_, riboflavin and vitamin B_6_ intake. No previous study has investigated the association between dietary intake and status in these interdependent B-vitamins, in the absence of mandatory folic acid fortification and excluding B-vitamin users, in a randomly selected representative sample of an adult Mediterranean population.

Our aims were to assess, in Mediterranean adults in Southern Catalonia, folate, vitamin B_12_, riboflavin and vitamin B_6_ intake and status, adherence to the Mediterranean diet and the association between Mediterranean diet adherence (MDA) and B-vitamin intake and status. Our focus is on the four B-vitamins that play key and interdependent roles within the one-carbon metabolic network and that are the most relevant for clinical decision-making and public health policy with regard to their roles in this process. We refer to the locally sourced foods in the Mediterranean region, using the recently coined “Km 0” term.

## 2 Methods

### 2.1 Study participants

The cross-sectional study, conducted between 1998 and 2002, included a representative sample of the adult populations of two Mediterranean towns (one situated 10 km inland and one on the coast) in Tarragona province, Northeast Spain. Previous publications have outlined the study's design and recruitment procedures (Berrocal-Zaragoza et al., 2009). In summary, a representative sample of 812 individuals aged 18–77, was randomly selected from town hall population records. Exclusion criteria included: B-vitamin users, and women planning a pregnancy or that were pregnant or lactating. Approval for the study was obtained from the Ethics Committees of Hospital Universitari Sant Joan de Reus and Fundació Jordi Gol i Gorina (ref.: CEIC June 30, 1998 and ref.: 03–03–20/3proj2, respectively).

### 2.2 Blood samples, B-vitamin status and polymorphism determination

Participants provided blood samples following an overnight fast. These samples were maintained at 4 °C until processing, which occurred within 2 h of collection. Plasma and whole blood aliquots, diluted 1:10 with a 1 % ascorbic acid solution for red blood cell folate (RBCF) determinations, and washed erythrocyte hemolysates for erythrocyte glutathione reductase activation coefficient (EGRAC) and erythrocyte aspartate aminotransferase activation coefficient (EASTAC), were stored at −80 °C until analysis. Plasma folate and RBCF concentrations were determined by microbiological assay (*Lactobacillus casei*) (Molloy and Scott, 1997), while plasma B_12_ concentrations were determined by *Lactobacillus leichmannii* (Kelleher and Broin, 1991). These analyses were conducted at Professors John Scott and Anne Molloys’ laboratory at Trinity College, Dublin, Ireland. Holotranscobalamin (holoTC) and fasting total homocysteine (tHcy) were determined by the Abbott IMx fluorescence polarization immunoassay (Abbott Laboratories Diagnostics Division) in the Central Laboratory in Hospital Sant Joan, Reus. EGRAC and EASTAC were determined as previously described (García-Minguillán et al., 2014), Plasma methylmalonic acid (MMA) was measured using gas chromatography-tandem mass spectrometry (GC-MS) and plasma creatinine was determined by the Jaffé reaction (Química Clínica Aplicada, SA). The *MTHFR* 677 C > T (rs1801133), *SLC19A1* 80 G > A (rs1051266), *MTRR* 66 A > G (rs1801394), *MTRR* 524 C > T (rs1532268), *MTR* 2756 A > G (rs1805087), *TCII* 776 C > G (rs1801198) and *TCII* 67 A > G (rs9606756) polymorphisms were determined on leukocyte-extracted. DNA by matrix-assisted laser desorption/ionization/time-of-flight MS (Meyer et al., 2004) as previously described (Bueno et al., 2016; García-Minguillán et al., 2014).

The Combined Indicator of Vitamin B_12_ (cB12) status, was calculated using the spreadsheets provided by Fedosov et al. (2015) applying the equation: cB12 = log_10_ [holoTC x B_12_/(MMA x tHcy)] – [3.79/1+(age/230)^2.6^], adjusting for plasma folate concentration.

### 2.3 Lifestyle, dietary habits and adherence to the mediterranean diet

Anthropometric measurements and lifestyle habits were recorded during a medical check-up by nurses and trained personnel. Participants recorded their dietary intake using a 3-day dietary record. They were instructed to document all consumed foods and beverages, including snacks, over two non-consecutive weekdays and one holiday, with portion sizes estimated using household measures. On completion of the records, participants answered specific questions regarding foods that they like most and least as well as foods that they consider they eat too much or too little of, as well as questions regarding their habitual appetite. They were also asked whether they usually eat at home and how often they eat out and for which meal. They were specifically asked regarding the frequency of vegetable and legume intake and cooking methods of these. Specifically, participants were not recruited between December 15th -Jan 15th to prevent recording the atypical diet during the traditional celebrations of this time of the year, around Europe. The 2 trained dietitians on the study team were responsible for interviewing the participants, validating, coding and inputting the dietary data. Participants submitted their completed 3-day dietary records by appointment for an interview with one of the dietitians. During this interview, the dietitian reviewed each record to verify and clarify the reported foods and quantities, using validated picture albums of food items and portions to verify portion sizes. Cooking methods and condiment use were confirmed, ambiguities clarified and participants were interrogated regarding missing items from the records and were also asked whether they had been in good health and whether the records reflected their usual diet, so that their food intake could be calculated to the maximum precision. Each record was then codified by the dietitian, and nutrient intake was calculated based on the French and Spanish Food Composition Tables (Favier et al., 1997; Mataix, 1995). Fortified breakfast cereal consumption was also recorded.

We used the European Food Safety Authority (EFSA) population reference intake (PRI), average requirement (AR) and tolerable upper level (UL) recommendations to estimate the prevalence of inadequacy for all of the vitamins and of excessive intake for vitamin B_6_, and folate (European Food Safety Authority (EFSA), 2017; European Food Safety Authority (EFSA), 2024). No UL has been defined for riboflavin or vitamin B_12_ (European Food Safety Authority (EFSA), 2024). Because the EFSA did not set a PRI and AR for vitamin B_12_, the US Food and Nutrition Board of the Institute of Medicine (IOM) (Institute of Medicine (US) Standing Committee on the Scientific Evaluation of Dietary Reference Intakes and its Panel on Folate, 1998) recommendations were used to estimate the prevalence of inadequacy as the recommended dietary allowance (RDA) and estimated average requirement (EAR).

Dietary folate equivalents (DFE) were calculated to account for the difference in bioavailability of folate from food is lower compared to folic acid added to fortified foods (Institute of Medicine (US) Standing Committee on the Scientific Evaluation of Dietary Reference Intakes and its Panel on Folate, 1998).

Elevated EGRAC (≥1.4) and EASTAC (≥1.6), were used as indicators of riboflavin (Powers et al., 2011) and vitamin B_6_ deficiency (Sauberlich et al., 1972), respectively. Folate deficiency was defined as plasma folate <7 nmol/L (Chanarin et al., 1986), and RBCF deficiency as RBCF <340 nmol/L (De Benoist, 2008; Selhub et al., 2008). We also used the cut-off values set by WHO to reduce the risk of NTDs, RBCF >906 nmol/L in women of childbearing age (World Health Organization). Deficiency in vitamin B_12_ and its functional markers were defined as plasma B_12_ < 148 pmol/L, holoTC <35 pmol/L, MMA >0.37 μmol/L, cB12 <-0.5, and tHcy >15 μmol/L (Green et al., 2017).

The Trichopoulou score was used to evaluate Mediterranean diet adherence (MDA) (Trichopoulou et al., 2003). Participants were assigned a value of 1 if their intake of components deemed beneficial (such as: all grains, fruits and nuts, vegetables, legumes, fish and the ratio of monounsaturated (MUFA) to saturated fatty acids (SFA)) was equal to or exceeded the study's population median. Conversely, individuals were assigned a value of 0 if their consumption of these beneficial foods was below the median. The consumption of components not considered beneficial (such as: meat and dairy products) equal to or below the median were assigned a value of 1, while those with consumption above the median were assigned a value of 0. Regarding alcohol intake, a value of 1 was assigned to subjects with moderate alcohol consumption based on Trichopoulou score categories (>10 and < 50 g/day for men, and >5 and < 25 g/day for women), and a value of 0 otherwise. Consequently, the total Trichopoulou score ranged from 0 (indicating minimal adherence to the Mediterranean diet) to 9 (representing maximal adherence).

### 2.4 Statistical analysis

For comparative purposes the population was stratified into 4 groups: women, aged ≤50 y; men, aged ≤50 y; women, aged >50 y and men, aged >50 y. The cut-off of 50 y was applied to clearly distinguish between adults of fertile age and ageing adults. Variable distribution and homogeneity of variances were evaluated using the Kolmogorov-Smirnov test and Levene's test, respectively, and variables with skewed distributions were natural log transformed to approach normality for the application of parametric tests. Quantitative variables were compared using the Student's t-test for normally distributed variables or the Mann-Whitney *U* test for non-normally distributed variables when comparing two groups. For four-group comparisons, ANOVA was applied to normally distributed variables, while the Kruskal-Wallis test was used for non-normally distributed variables, with post-hoc Bonferroni correction for multiple comparisons. Categorical variables were compared using the Chi-square test.

Associations between dietary intake (riboflavin, vitamin B_6_, folate and vitamin B_12_ intake) and EGRAC, EASTAC, plasma folate, RBCF, plasma B_12_, holoTC, MMA, cB12 and tHcy status were analysed using multiple linear regression analysis. Interactions between biomarker status and genotype, as well as between dietary intake and smoking, were explored. For the regression models predicting tHcy, we tested alternative predictors by replacing plasma B_12_ with holoTC or with MMA to assess which provided a better fit. Participants with extreme energy intake values (<800 kcal/day or >4000 kcal/day) were identified, and a sensitivity analysis excluding these participants was performed. Additionally, a sensitivity analysis excluding participants on proton pump inhibitor (PPI) medication was performed. To assess potential sex-specific differences, regression models were repeated stratifying by sex. Additional analyses of plasma B_12_, holoTC, and MMA were conducted separately, first by stratifying based on the median plasma B_12_ level and then by sex, to assess whether these groupings influenced the observed associations. Models were adjusted for potential confounders including age, sex, body mass index (BMI), socioeconomic status, smoking, at-risk alcohol consumers, creatinine and energy intake.

Differences between quartile groups of Mediterranean diet adherence, were assessed using the ANOVA test for normally distributed continuous variables, and the Kruskal-Wallis test for variables with nonparametric distributions with Post-hoc Bonferroni correction for multiple comparisons. Categorical variables were compared using the Chi-square test. Spearman correlation coefficients were calculated to evaluate the correlation between dietary intake and blood biomarkers and then corrected for Bonferroni correction for multiple comparisons. 57 participants were excluded from the tHcy analysis due to their samples being processed 2–4 h post-collection. All statistical analyses were performed using SPSS version 29.0 (SPSS Inc., Chicago, IL), with statistical significance set at *P* < 0.05.

## 3 Results

A total of 812 men and women were initially included. Of these, 24 were excluded for using B-vitamins, 43 did not return dietary intake questionnaires, 2 had altered renal function (plasma creatinine >97 mmol/L for women and >124 mmol/L for men) and 3 participants received vitamin B_12_ injections. The 740 participants included in the current analysis consisted of 388 women and 352 men (Supplementary Figure S1).

The demographic and genetic characteristics of the study participants are reported in Table 1. Higher BMI was observed in the >50 y age groups compared to ≤50 y for both women and men. The highest prevalence of low socioeconomic status was observed in women >50 y and the lowest in men <50 y. More of the ≤50 y age group smoked compared to the >50 y age group, for both sexes. At-risk alcohol consumption was higher in men compared to women in both young and old participants. The prevalence of commercial breakfast cereal consumption was 7.2 %, and it was very low in men >50 y. Globally the prevalence of the homozygote variant genotypes of the investigated polymorphisms was highest for *MTRR* 66 A > G (24.5 %), and lowest for *TCII* 67 A > G (1.1 %).

**Table 1:** Demographic and genetic profile of all participants and subgroups stratified by gender and age.

		Overall n = 740	Women ≤50 y n = 266	Men ≤50 y n = 236	Women >50 y n = 122	Men >50 y n = 116	*P-*value
Demographic information							
Age (years)^a^		41.0 (30.0, 54.0)	34.0 (26.0, 41.3)	34.0 (26.0, 42.0)	60.0 (54.0, 69.0)	60.5 (55.0, 67.0)	<0.001
BMI (kg/m^2^)^a^		26.7 (23.2, 30.0)	23.6 (21.3, 27.2)	26.4 (23.8, 29.3)	29.7 (26.9, 32.9)	28.9 (26.4, 31.8)	<0.001
Socioeconomic status, low studies and profession^b^		37.6 (34.2, 41.1)	30.5 (25.2, 36.2)	11.9 (8.3, 16.6)	82.0 (74.2, 87.8)	59.5 (50.4, 68.0)	<0.001
Active smoking^b^		32.6 (29.3, 36.0)	41.7 (36.0, 47.7)	41.5 (35.4, 47.9)	6.6 (3.4, 12.4)	20.7 (14.3, 28.9)	<0.001
Alcohol intake, prevalence of high-risk intake^b^^,^^c^		15.1 (12.7, 17.9)	4.5 (2.6, 7.7)	22.0 (17.2, 27.7)	6.6 (3.4, 12.4)	34.5 (26.5, 43.5)	<0.001
Proton pump inhibitor use^b^		1.2 (0.6, 2.3)	0.4 (0.1, 2.1)	1.3 (0.4, 3.7)	0.8 (0.1, 4.5)	3.4 (1.3, 8.5)	0.195
Breakfast cereal consumption^b^		7.2 (5.5, 9.3)	9.4 (6.4, 13.5)	8.9 (5.9, 13.2)	5.7 (2.8, 11.4)	0.0 (0.0, 3.2)	0.006
**Genetic polymorphisms**							
*MTHFR* 677 C > T^b^	CC	35.9 (32.5, 39.4)	33.2 (27.8, 39.1)	37.4 (31.5, 43.8)	38.7 (30.4, 47.6)	36.0 (27.7, 45.1)	0.711
	CT	46.0 (42.6, 49.7)	50.4 (44.4, 56.4)	43.0 (36.8, 49.4)	44.5 (35.9, 53.5)	43.9 (35.1, 53.0)	
	TT	18.1 (15.5, 21.0)	16.4 (12.4, 21.4)	19.6 (15.0, 25.1)	16.8 (11.2, 24.5)	20.2 (13.8, 28.5)	
*SLC19A1* 80 G > A^b^	GG	25.5 (22.5, 28.8)	21.8 (17.2, 27.1)	28.5 (23.1, 34.6)	25.2 (18.3, 33.7)	28.1 (20.6, 36.9)	0.154
	GA	50.7 (47.1, 54.3)	52.3 (46.3, 58.3)	52.8 (46.4, 59.1)	51.3 (42.4, 60.1)	42.1 (33.4, 51.3)	
	AA	23.3 (20.9, 27.1)	26.0 (21.0, 31.6)	18.7 (14.3, 24.2)	23.5 (16.8, 31.9)	29.8 (22.2, 38.8)	
*MTRR* 66 A > G^b^	AA	26.4 (23.4, 29.8)	28.2 (23.1, 34.0)	28.1 (22.7, 34.1)	24.4 (17.5, 32.8)	21.1 (14.6, 29.4)	0.689
	AG	49.0 (45.4, 52.7)	49.2 (43.2, 55.3)	46.8 (40.5, 53.2)	47.9 (39.1, 56.8)	54.4 (45.2, 63.2)	
	GG	24.5 (21.5, 27.8)	22.5 (17.9, 28.0)	25.1 (20.0, 31.0)	27.7 (20.5, 36.4)	24.6 (17.6, 33.2)	
*MTRR* 524 C > T^b^	CC	40.0 (36.5, 43.6)	42.4 (36.5, 48.4)	37.0 (31.1, 43.4)	42.9 (34.3, 51.8)	37.7 (29.4, 46.9)	0.882
	CT	46.8 (43.3, 50.5)	44.7 (38.8, 50.7)	49.8 (43.4, 56.1)	43.7 (35.1, 52.7)	49.1 (40.1, 58.2)	
	TT	13.2 (10.9, 15.8)	13.0 (9.4, 17.6)	13.2 (9.5, 18.1)	13.4 (8.4, 20.7)	13.2 (8.1, 20.6)	
*MTR* 2756 A > G^b^	AA	71.1 (67.7, 74.3)	73.3 (67.6, 78.3)	68.5 (62.3, 74.1)	73.9 (65.4, 81.0)	68.4 (59.4, 76.2)	0.861
	AG	26.2 (23.1, 29.5)	24.0 (19.3, 29.6)	28.9 (23.5, 35.0)	23.5 (16.8, 31.9)	28.1 (20.6, 36.9)	
	GG	2.7 (1.8, 4.2)	2.7 (1.3, 5.4)	2.6 (1.2, 5.5)	2.5 (0.9, 7.2)	3.5 (1.4, 8.7)	
*TCII* 776 C > G^b^	CC	33.8 (30.5, 37.3)	29.8 (24.6, 35.6)	33.6 (27.9, 39.9)	34.5 (26.5, 43.4)	43.0 (34.3, 52.2)	0.182
	CG	46.0 (42.4, 49.7)	51.5 (45.5, 57.5)	46.0 (39.7, 52.3)	42.9 (34.3, 51.8)	36.8 (28.6, 46.0)	
	GG	20.1 (17.4, 23.2)	18.7 (14.4, 23.9)	20.4 (15.8, 26.0)	22.7 (16.1, 31.0)	20.2 (13.8, 28.5)	
*TCII* 67 A > G^b^	AA	79.9 (76.8, 82.6)	80.5 (75.3, 84.9)	80.0 (74.4, 84.6)	75.6 (67.2, 82.5)	82.5 (74.4, 88.3)	0.390
	AG	19.0 (16.4, 22.0)	17.9 (13.8, 23.0)	18.3 (13.9, 23.7)	24.4 (17.5, 32.8)	17.5 (11.7, 25.6)	
	GG	1.1 (0.6, 2.1)	1.5 (0.6, 3.9)	1.7 (0.7, 4.3)	0.0 (0.0, 3.1)	0.0 (0.0, 3.3)	

The Kruskal-Wallis test and chi-square tests were used to compare differences between the subgroups. Bonferroni post hoc correction of *P*-values for multiple comparisons was performed.

Abbreviations. BMI: body mass index; MTHFR: methylenetetrahydrofolate reductase; MTR: methionine transferase; MTRR: methionine transferase reductase; SLC19A1: solute carrier family 19A, member 1; TCII: transcobalamin II; y: years.

a Median (25th, 75th percentile).

b Percentage (95 % confidence interval).

c Prevalence of high-risk intake >24 g/day in men and >16 g/day in women.

B-vitamin dietary intake and status are reported in Table 2. Men ≤50 y and >50 y had higher energy intakes compared to women in the same age group. Within each sex, women ≤50 y had higher energy intakes than women >50 y, and men ≤50 y also had higher energy intakes than men >50 y. Men ≤50 y had higher intakes of riboflavin, vitamin B_6_, energy adjusted vitamin B_6_, folate and vitamin B_12_ compared to women of the same age group. A similar pattern was observed in men >50 y compared to women >50 y for all vitamins, with the exception of riboflavin, for which the difference was not statistically significant. Same sex comparisons showed that women ≤50 y had higher vitamin B_6_ intake and lower energy adjusted folate intake compared to women >50 y, and men ≤50 y had higher riboflavin intake, and lower energy adjusted folate intakes compared to men >50 y. No differences in energy-adjusted intakes of riboflavin and vitamin B_12_ occurred between the different groups. However, in participants ≤50 y energy-adjusted vitamin B_6_ intake was higher in men than in women, and comparing participants ≤50 y with those >50 y, energy-adjusted folate intake was lower in younger compared to older women and in younger compared to older men.

**Table 2:** Dietary intake and B-vitamin status of all participants and subgroups stratified by gender and age.

	Overall n = 740	Women ≤50 y n = 266	Men ≤50 y n = 236	Women >50 y n = 122	Men >50 y n = 116
Dietary intake					
Energy (kcal/day)^a^	2125.0 (1676.6, 2607.5)	1913.6 (1593.2, 2245.7)	2638.6 (2261.3, 3119.0)^i^	1585.1 (1340.2, 1941.2)^k^	2239.2 (1844.7, 2589.3)^j^, ^l^
Riboflavin intake (mg/day)^a^	1.7 (1.4, 2.1)	1.6 (1.3, 1.9)	1.9 (1.6, 2.3)^i^	1.5 (1.2, 1.7)	1.6 (1.3, 2)^l^
Energy adjusted riboflavin intake^a^	1.8 (1.7, 2.0)	1.8 (1.7, 1.9)	1.9 (1.7, 2.0)	1.8 (1.7, 1.9)	1.8 (1.7, 1.9)
Vitamin B_6_ intake (mg/day)^a^	1.8 (1.4, 2.3)	1.6 (1.3, 1.9)	2.2 (1.9, 2.7)^i^	1.5 (1.3, 1.8)^k^	2.0 (1.6, 2.5)^j^
Energy adjusted vitamin B_6_ intake^a^	1.9 (1.6, 2.2)	1.8 (1.6, 2.1)	1.9 (1.6, 2.2)^i^	1.9 (1.7, 2.1)	2.0 (1.6, 2.4)
Folate intake (μg/day DFE)^a^^,^^c^	279.3 (214.5, 360.8)	247.4 (196.7, 337.9)	295.7 (223.0, 369.7)^i^	269.9 (215.5, 353.3)	318.1 (258.3, 393.7)^j^
Energy adjusted folate intake^a^^,^^c^	281.8 (220.6, 362.2)	267.8 (208.2, 349.4)	259.3 (205.7, 345.6)	307.0 (259.4, 396.1)^k^	303.5 (255.0, 391.4)^l^
Vitamin B_12_ intake (μg/day)^a^	4.4 (3, 6.4)	3.7 (2.8, 5.5)	5.3 (3.9, 7.5)^i^	3.4 (2.3, 5.4)	4.8 (3.2, 7.4)^j^
Energy adjusted vitamin B_12_ intake^a^	4.3 (3.2, 6.1)	4.2 (3.3, 5.9)	4.5 (3.4, 6.5)	4.1 (3.3, 5.7)	4.8 (2.9, 6.7)
**Low and high dietary intake**					
Riboflavin intake < PRI^b^^,^^d^	43.9 (40.4, 47.5)	48.1 (42.2, 54.1)	28.0 (22.6, 34.0)^i^	60.7 (51.8, 68.9)^k^	49.1 (40.2, 58.1)^l^
Riboflavin intake < AR^b^^,^^e^	20.1 (17.4, 23.2)	22.9 (18.3, 28.3)	8.9 (5.9, 13.2)^i^	32.8 (25.1, 41.5)^k^	23.3 (16.5, 31.7)^l^
Riboflavin intake > UL^b^^,^^f^	–	–	–	–	–
Vitamin B_6_ intake < PRI^b^^,^^d^	38.0 (34.5, 41.5)	47.4 (41.4, 53.4)	17.4 (13.1, 22.7)^i^	64.8 (55.9, 72.7)^k^	30.2 (22.6, 39.1)^j^^,^^l^
Vitamin B_6_ intake < AR^b^^,^^e^	19.6 (16.9, 22.6)	24.1 (19.3, 29.5)	10.2 (6.9, 14.7)^i^	32.0 (24.4, 40.7)	15.5 (10.0, 23.2)^j^^,^^l^
Vitamin B_6_ intake > UL^b^^,^^f^	0.0 (0.0, 0.5)	0.0 (0.0, 1.4)	0.0 (0.0, 1.6)	0.0 (0.0, 3.1)	0.0 (0.0, 3.2)
Folate intake < PRI^b^^,^^d^	65.8 (62.3, 69.1)	73.3 (67.7, 78.3)	62.3 (56.0, 68.2)^i^	68.9 (60.2, 76.4)	52.6 (43.6, 61.4)^j^
Folate intake < AR^b^^,^^e^	39.5 (36.0, 43.0)	51.1 (45.1, 57.1)	34.3 (28.6, 40.6)^i^	41.0 (32.7, 49.9)	21.6 (15.0, 29.9)^j^^,^^l^
Folate intake > UL^b^^,^^f^	0.0 (0.0, 0.5)	0.0 (0.0, 1.4)	0.0 (0.0, 1.6)	0.0 (0.0, 3.0)	0.0 (0.0, 3.2)
Vitamin B_12_ intake < RDA^b^^,^^g^	13.2 (11.0, 15.9)	15.0 (11.2, 19.8)	3.8 (2.0, 7.1)^i^	29.5 (22.1, 38.1)^k^	11.2 (6.7, 18.2)^j^^,^^l^
Vitamin B_12_ intake < EAR^b^^,^^h^	6.5 (4.9, 8.5)	6.4 (4.0, 10.0)	1.3 (0.4, 3.7)^i^	16.4 (10.9, 24.0)^k^	6.9 (3.5, 13.0)^j^^,^^l^
Vitamin B_12_ intake > UL^b^^,^^f^	–	–	–	–	–
**B-vitamin status**					
EGRAC^a^	1.3 (1.2, 1.5)	1.4 (1.2, 1.6)	1.4 (1.2, 1.5)	1.2 (1.1, 1.4)^k^	1.3 (1.2, 1.4)^l^
EASTAC^a^	1.6 (1.5, 1.8)	1.6 (1.5, 1.8)	1.6 (1.5, 1.8)	1.6 (1.5, 1.8)	1.6 (1.4, 1.7)
Plasma folate (nmol/L)^a^	11.1 (7.9, 16.2)	10.4 (7.5, 15.0)	9.3 (6.6, 12.8)^i^	17.0 (10.6, 24.3)^k^	14.6 (10.7, 19.1)^j^^,^^l^
RBCF (nmol/L)^a^	794.2 (615.7, 1009.3)	712.8 (541.0, 912.0)	748.8 (592.4, 968.1)	932.6 (765.9, 1169.9)^k^	936.2(721.7, 1193.1)^l^
Plasma B_12_ (pmol/L)^a^	347.5 (274.2, 439.1)	329.5 (261.6, 432.9)	350.8 (274.7, 432.8)	388.2 (320.3, 474.6)^k^	336.7 (264.5, 446.5)^j^
Plasma holoTC (pmol/L)^a^	78.7 (58.1, 105.5)	78.8 (56.6, 103.0)	77.6 (61.3, 107.9)	78.4 (56.3, 108.6)	80.8 (56.6, 106.5)
Plasma MMA (μmol/L)^a^	0.1 (0.1, 0.2)	0.1 (0.1, 0.2)	0.1 (0.1, 0.2)	0.1 (0.1, 0.2)	0.1 (0.1, 0.2)
cB12^a^	0.7 (0.4, 0.9)	0.7 (0.4, 0.9)	0.6 (0.4, 0.8)	0.7 (0.5, 1.0)	0.6 (0.4, 0.8)
Plasma tHcy (μmol/L)^a^	9.3 (7.8, 11.1)	8.3 (6.9, 9.9)	9.8 (8.4, 11.4)^i^	9.3 (7.8, 11.1)^k^	10.3 (9.2, 12.3)^j^
**Low B-vitamin status**					
EGRAC ≥1.4^b^	35.9 (32.5, 39.4)	45.0 (39.1, 51.1)	39.7 (33.6, 46.1)	21.7 (15.2, 29.9)^k^	21.9 (15.3, 30.4)^l^
EASTAC ≥1.6^b^	56.0 (52.4, 59.6)	60.5 (54.4, 66.2)	57.6 (51.1, 63.8)	52.1 (43.2, 61.0)	46.5 (37.6, 55.6)^l^
Plasma folate <7 nmol/L^b^	18.6 (16.0, 21.6)	21.4 (16.9, 26.7)	27.5 (22.2, 33.6)	4.9 (2.3, 10.3)^k^	8.6 (4.7, 15.1)^l^
Plasma folate <10 nmol/L^b^	41.4 (37.9, 44.9)	46.2 (40.3, 52.2)	58.5 (52.1, 64.6)^i^	18.9 (12.9, 26.7)^k^	19.0 (12.9, 27.0)^l^
RBCF <340 nmol/L^b^	1.5 (0.8, 2.6)	2.3 (1.0, 4.8)	2.1 (0.9, 4.9)	0.0 (0.0, 3.1)	0.0 (0.0, 3.2)^l^
RBCF <906 nmol/L^b^	63.2 (59.7, 66.6)	74.1 (68.5, 79.0)	69.9 (63.8, 75.4)	43.4 (35.0, 52.3)^k^	45.7 (36.9, 54.7)^l^
Plasma B_12_ < 148 pmol/L^b^	1.6 (0.9, 2.8)	2.3 (1.0, 4.8)	0.8 (0.2, 3.0)	2.5 (0.8, 7.0)	0.9 (0.2, 4.7)
Plasma B_12_ < 221 pmol/L^b^	9.5 (7.6, 11.8)	12.8 (9.3, 17.3)	8.1 (5.2, 12.2)	5.7 (2.8, 11.4)^k^	8.6 (4.7, 15.1)
Plasma holoTC <35 pmol/L^b^	3.8 (2.7, 5.5)	4.6 (2.7, 7.9)	3.0 (1.5, 6.1)	3.3 (1.3, 8.1)	4.3 (1.9, 9.7)
Plasma MMA >0.37 μmol/L^b^	1.2 (0.6, 2.3)	1.1 (0.4, 3.3)	2.1 (0.9, 4.9)	0.8 (0.1, 4.5)	0.0 (0.0, 3.2)
cB12 <-0.5^b^	0.7 (0.3, 1.6)	0.4 (0.1, 2.1)	0.9 (0.2, 3.0)	1.6 (0.5, 5.8)	0.0 (0.0, 3.2)
Plasma tHcy >15 μmol/L^b^	4.5 (3.2, 6.4)	2.9 (1.4, 5.8)	7.7 (4.9, 12.0)^i^	1.8 (0.5, 6.3)	4.7 (2.0, 10.5)

The Mann-Whitney test and chi-square tests were used to compare differences between the subgroups. Post-hoc Bonferroni correction of *P*-values was performed to correct for multiple comparisons. Different superscripts letters indicate significant differences between groups.

Abbreviations. AR: average requirement; B_12_: cobalamin; B_6_: pyridoxine; BMI: body mass index; cB12: combined indicator of vitamin B12 status; DFE: dietary folate equivalents; EAR: estimated average requirement; EASTAC: erythrocyte aspartate aminotransferase activation coefficient; EGRAC: erythrocyte glutathione reductase activation coefficient; holoTC: holotranscobalamin; MMA: methylmalonic acid; PRI: population reference intake; RBCF: red blood cell folate; RDA: recommended dietary allowance; tHcy: fasting total homocysteine; UL: upper intake level; y: year.

a Median (25th, 75th percentile).

b Percentage (95 % confidence interval).

c μg/day of dietary folate equivalents (1 μg of DFE = 1 μg of food folate = 0.6 μg of folic acid from fortified foods and supplements) (Institute of Medicine (US) Standing Committee on the Scientific Evaluation of Dietary Reference Intakes and its Panel on Folate, 1998).

d Population reference intake (PRI) (European Food Safety Authority (EFSA), 2017): riboflavin: 1.6 mg/day; vitamin B_6_: women: 1.6 mg/day, and men: 1.7 mg/day, and folate: 330 μg/day of DFE.

e Average requirement (AR) (European Food Safety Authority (EFSA), 2017): riboflavin: 1.3 mg/day; vitamin B_6_: women: 1.3 mg/day, and men: 1.5 mg/day, and folate: 250 μg/day of DFE.

f Upper level (UL) (European Food Safety Authority (EFSA), 2024): riboflavin: no adequate data to derive a UL; vitamin B_6_: 25 mg/day; folate: 1000 μg/day of DFE; vitamin B_12_: no defined adverse effects.

g Recommended dietary allowance (RDA) (Institute of Medicine (US) Standing Committee on the Scientific Evaluation of Dietary Reference Intakes and its Panel on Folate, 1998): vitamin B_12_: 2.4 μg/day.

h Estimated average requirement (EAR) (Institute of Medicine (US) Standing Committee on the Scientific Evaluation of Dietary Reference Intakes and its Panel on Folate, 1998): vitamin B_12_: 2.0 μg/day.

i women ≤50 y vs. men ≤50.

j women >50 y vs. men >50 y.

k women ≤50 vs. women >50 y.

l men ≤50 y vs. men >50 y.

Globally, the proportion of participants that did not reach the average intake requirement was 20.1 %, 19.6 %, and 39.5 % for riboflavin, vitamin B_6_, and folate, respectively. In the case of vitamin B_12_, only 6.5 % of the participants did not reach the EAR. In women of both age groups, the prevalence of intake for the 4 B-vitamins below the AR and EAR was higher than in men. The prevalence of riboflavin and vitamin B_6_ intake below the AR and of vitamin B_12_ intake < EAR was higher in participants >50 y compared to ≤50 y. However, in the case of folate intake the prevalence of participants not meeting the AR was higher participants ≤50 y compared to >50 y. No participant exceeded the tolerable upper level (UL) for the vitamins with a defined UL, vitamin B_6_ and folate.

Differences were observed in the vitamin blood biomarkers between sex and age groups. EGRAC was lower in the >50 y versus the ≤50 y group for both sexes. However, it did not differ between sexes. The median EASTAC value for the study participants and the groups, was above the cut-off point for vitamin B_6_ status deficiency, indicating a high prevalence of deficiency. Plasma folate status was higher in women compared to men in both age groups and in the >50 y versus the ≤50 y groups. Similarly, RBCF status was higher in the >50 y versus the ≤50 y groups but did not differ between men and women of the same age group. Plasma B_12_ status was higher in women >50 y compared to women ≤50 y. Plasma holoTC and MMA, as well as cB12 did not differ aming any of the groups. Plasma tHcy status was higher in men compared to women, in all of the age groups. Deficiency in the 4 vitamins occurred in all of the groups. EGRAC ≥1.4, indicating riboflavin status deficiency, was more prevalent in women and men ≤50 y compared to >50 y but did not differ between sexes. EASTAC ≥1.6, indicating vitamin B_6_ status deficiency, occurred in 56.0 % of the participants. It was more prevalent in men ≤50 y compared to >50 y but did not differ between sexes or between the younger and older age groups in women. The prevalence of plasma folate deficiency (<7 nmol/L) was 18.6 %. It was higher in the ≤50 y versus >50 y age group, both in women and men and this was also true for plasma folate <10 nmol/L. RBCF <340 nmol/L, indicating folate deficiency, occurred in 1.5 % of the participants and was confined to those ≤50 y. The majority of the women ≤50 y did not meet the WHO recommended RBCF of 906 nmol/L, the threshold value to prevent NTDs (74.1 %). Plasma B_12_ < 148 pmol/L was observed in 1.6 % of the participants and did not differ between sex or age groups. Plasma holoTC <35 pmol/L, MMA >0.37 μmol/L, and cB12 <-0.5 varied from 3.0 to 4.6 %, 0.0–2.1 % and 0.0–1.6 % respectively across the different sex and age groups, and did not differ among them. Plasma tHcy >15 μmol/L was prevalent in 4.5 % of the study participants, and occurred in more men ≤50 y compared to women of the same age group.

Differences in B vitamin intake and blood biomarkers between breakfast cereal consumers and non-consumers are shown in Supplementary Table S1. Breakfast cereal consumers had higher intakes of riboflavin, vitamin B_6_, and folate compared to non-consumers. Furthermore, breakfast cereal consumers had higher plasma folate status compared to non-consumers.

Additional analyses comparing participants living in inland versus coastal areas revealed some differences in B-vitamin intake and status (Supplementary Table S2). Inland participants reported slightly lower vitamin B_6_ intake and exhibited higher EGRAC and EASTAC values, suggesting lower riboflavin and vitamin B_6_ status, respectively. Conversely, inland participants had higher RBCF and lower tHcy status compared with those from coastal areas. Socioeconomic differences were also observed, with a higher prevalence of low education level and profession in coastal participants. We also explored potential seasonal variations in energy intake and vitamin intake across winter, spring, summer, and autumn. The analysis showed that average energy intake or folate, vitamin B_12_, riboflavin, and vitamin B_6_ intakes did not differ (all *P* > 0.05) between the four seasons of the year (data not shown).

The results of multiple linear regression analyses exploring the associations between, dietary intake, blood biomarkers, and B-vitamin status are reported in Table 3. Nine participants with implausible energy intake according to their returned food diaries (one below 800 kcal/day, and eight above 4000 kcal/day) were excluded from the analyses. The results show that the fully adjusted models predicting EGRAC and EASTAC status accounted for 9.5 % and 5.3 % of their variance, respectively. Plasma B_12_ was the strongest, and only significant, predictor of EGRAC, while plasma folate was the strongest, and only significant, predictor of EASTAC. Plasma folate was best predicted by folate intake, followed by the *MTHFR* 677 TT genotype, *MTHFR* 677CT genotype, plasma B_12_, and then EASTAC, in a model explaining 31.0 % of its variance. In the case of RBCF, the *MTHFR* 677 TT genotype was the strongest predictor, followed by folate intake, plasma B_12_, *SLC19A1* 80AA genotype, EASTAC, and the *MTHFR* 677CT genotype. This model explained 31.0 % of the variance. Low variance was predicted in the plasma B_12_ models, which was mainly predicted by EGRAC, followed by vitamin B_12_ intake. Addition of genotypes for known polymorphisms that affect vitamin B_12_ transport or its role in 1 carbon metabolism (methionine transferase reductase (*MTRR*) 66 A > G (rs1801394) (7), methionine transferase reductase (*MTRR*) 524 C > T (rs1532268) (8), methionine transferase (*MTR*) 2756 A > G (rs1805087) (9), transcobalamin II (*TCII*) 776 C > G (rs1801198) (10) or transcobalamin II (*TCII*) 67 A > G (rs9606756) (11)) did not improve the overall predictability of the models. None of the models testing predictors for plasma holoTC, MMA or the vitamin B_12_ status indicator, cB12, were significant. The plasma tHcy model 1 explained 15.1 % of the variance and dietary vitamin B_6_ intake was negatively associated with tHcy. Inclusion of the genotypes (fully adjusted model) improved the overall model, and the *MTHFR* 677 TT genotype was the strongest predictor, followed by plasma B_12_ and plasma folate, in a model explaining 30.5 % of the variance. The overall model that included the genotypes in the vitamin intake models explained 24.9 % of the variability (data not shown). Nine participants reported proton pump inhibitor use but none of them had vitamin B_12_ deficiency. However, sensitivity analyses excluding them from the models predicting plasma B_12_ status were performed. The models were only marginally affected and the results and conclusions were the same. Therefore, all participants were included in the final models.

**Table 3:** Multiple linear regression analysis exploring associations between blood biomarkers and B-vitamin status in all participants.

Biomarker	Model		β standardized coefficient	B unstandardized coefficient	95.0 % confidence interval	*P*-value	R^2^x100
**EGRAC**	**1**	Riboflavin intake (mg/day)	0.029	1.202x10^−4^	−1.823x10^−4^, 4.226x10^−4^	0.436	6.3∗∗∗
	**2**	Plasma B_12_ (pmol/L)	−0.145	−7.528x10^−5^	−1.123x10^−4^, −3.824x10^−5^	<0.001	9.0∗∗∗
		Plasma folate (nmol/L)	−0.068	−0.001	−1.276x10^−3^, 9.213x10^−5^	0.090	
		Riboflavin intake (mg/day)	0.037	1.558x10^−4^	−1.440x10^−4^, 4.557x10^−4^	0.308	
		EAST activation coefficient	−0.020	−0.006	−0.029, 0.016	0.581	
	**3**	Plasma B_12_ (pmol/L)	−0.145	−7.510x10^−5^	−1.121x10^−4^, −3.807x10^−5^	<0.001	9.5∗∗∗
		Plasma folate (nmol/L)	−0.077	−0.001	−0.001, 1.673x10^−5^	0.056	
		*MTHFR6*77 TT vs. CC	−0.076	−0.014	−0.028, 0.001	0.065	
		Riboflavin intake (mg/day)	0.036	1.500x10^−4^	−1.498x10^−4^, 4.497x10^−4^	0.326	
		EAST activation coefficient	−0.020	−0.006	−0.028, 0.016	0.589	
		*MTHFR6*77 CT vs. CC	−0.017	−0.002	−0.014, 0.009	0.671	
**EASTAC**	**1**	Vitamin B_6_ intake (mg/day)	−0.090	−0.008	−0.017, 0.001	0.094	4.1∗∗∗
	**2**	Vitamin B_6_ intake (mg/day)	−0.091	−0.008	−0.018, 0.002	0.099	5.3∗∗∗
		Plasma folate (nmol/L)	−0.087	−0.001	−0.001, 4.035x10^−5^	0.036	
		Plasma B_12_ (pmol/L)	0.060	2.546x10^−5^	−5.780x10^−6^, 5.669x10^−5^	0.110	
		EGR activation coefficient	−0.054	−0.013	−0.030, 0.005	0.165	
**Plasma folate**	**1**	Folate intake (μg/day)	0.309	0.001	4.763x10^−4^, 0.001	<0.001	27.8∗∗∗
	**2**	Folate intake (μg/day)	0.301	0.001	4.593x10^−4^, 0.001	<0.001	28.9∗∗∗
		EAST activation coefficient	−0.070	−0.073	−0.138, −0.007	0.031	
		Plasma B_12_ (pmol/L)	0.069	1.200x10^−4^	9.438x10^−6^, 2.305x10^−4^	0.033	
		EGR activation coefficient	−0.037	−0.036	−0.099, 0.027	0.266	
	**3**	Folate intake (μg/day)	0.300	0.001	4.577x10^−4^, 0.001	<0.001	31.0∗∗∗
		*MTHFR6*77 TT vs. CC	−0.146	−0.089	−0.132, −0.047	<0.001	
		*MTHFR6*77 CT vs. CC	−0.083	−0.039	−0.072, −0.006	0.020	
		Plasma B_12_ (pmol/L)	0.069	1.193x10^−4^	9.919x10^−6^, 2.288x10^−4^	0.033	
		EAST activation coefficient	−0.068	−0.070	−0.135, −0.005	0.034	
		EGR activation coefficient	−0.044	−0.042	−0.105, −0.005	0.191	
		*SLC19A1*80 AA vs. GG	−0.041	−0.023	−0.065, 0.019	0.290	
		*SLC19A1*80 GA vs. GG	0.030	−0.014	−0.022, 0.050	0.438	
**RBCF**	**1**	Folate intake (μg/day)	0.201	2.779x10^−4^	1.772x10^−4^, 3.785x10^−4^	<0.001	19.2∗∗∗
	**2**	Folate intake (μg/day)	0.196	2.704x10^−4^	1.697x10^−4^, 3.712x10^−4^	<0.001	22.0∗∗∗
		Plasma B_12_ (pmol/L)	0.147	1.775x10^−4^	9.683x10^−5^, 2.582x10^−4^	<0.001	
		EAST activation coefficient	−0.092	−0.066	−0.114, −0.018	0.012	
		EGR activation coefficient	0.003	0.002	−0.044, 0.048	0.999	
	**3**	*MTHFR6*77 TT vs. CC	−0.323	−0.138	−0.167, −0.108	<0.001	31.0∗∗∗
		Folate intake (μg/day)	0.183	2.525x10^−4^	1.566x10^−4^, 3.484x10^−4^	<0.001	
		Plasma B_12_ (pmol/L)	0.144	1.745x10^−4^	9.832x10^−5^, 2.507x10^−4^	<0.001	
		*SLC19A1*80 AA vs. GG	−0.115	−0.044	−0.074, −0.015	0.003	
		EAST activation coefficient	−0.087	−0.063	−0.108, −0.017	0.007	
		*MTHFR6*77 CT vs. CC	−0.086	−0.028	−0.051, −0.005	0.016	
		*SLC19A1*80 GA vs. GG	−0.048	−0.016	−0.041, 0.009	0.216	
		EGR activation coefficient	−0.011	−0.008	−0.051, 0.036	0.733	
**Plasma B_12_**	**1**	Vitamin B_12_ intake (μg/day)	0.154	0.005	0.002, 0.007	<0.001	3.2∗∗
	**2**	EGR activation coefficient	−0.153	−0.099	−0.147, −0.051	<0.001	6.1∗∗∗
		Vitamin B_12_ intake (μg/day)	0.137	0.004	0.002, 0.006	<0.001	
		Plasma folate (nmol/L)	0.074	0.001	−1.104x10^−4^, 0.003	0.067	
		EAST activation coefficient	0.038	0.026	−0.025, 0.077	0.310	
	**3**	EGR activation coefficient	−0.155	−0.100	−0.149, −0.051	<0.001	6.6∗∗
		Vitamin B_12_ intake (μg/day)	0.141	0.004	0.002, 0.007	<0.001	
		Plasma folate (nmol/L)	0.079	0.002	−4.683x10^−5^, 0.003	0.057	
		*MTRR5*24 CT vs. CC	−0.053	−0.017	−0.042, 0.008	0.181	
		EAST activation coefficient	0.038	0.027	−0.025, 0.079	0.311	
		*TCII6*7 GG vs. AA	−0.026	−0.040	−0.151, 0.072	0.485	
		*MTRR6*6 AG vs. AA	−0.025	−0.008	−0.036, 0.020	0.581	
		*MTR2*756 GG vs. AA	0.024	0.023	−0.048, 0.094	0.525	
		*TCII6*7 AG vs. AA	0.008	0.003	−0.027, 0.033	0.832	
		*TCII7*76 GG vs. CC	−0.007	−0.003	−0.036, 0.030	0.872	
		*MTRR5*24 TT vs. CC	0.006	0.003	−0.034, 0.040	0.875	
		*MTRR6*6 GG vs. AA	0.005	0.002	−0.031, 0.034	0.914	
		*TCII7*76 CG vs. CC	−0.005	−0.001	−0.028, 0.025	0.914	
		*MTR2*756 AG vs. AA	0.002	0.001	−0.026, 0.027	0.951	
**Plasma holoTC**^a^	**1**	–	–	–	–	–	1.3^NS^
	**2**	–	–	–	–	–	1.5^NS^
	**3**	–	–	–	–	–	3.3^NS^
**Plasma MMA**^a^	**1**	–	–	–	–	–	1.7^NS^
	**2**	–	–	–	–	–	1.9^NS^
	**3**	–	–	–	–	–	3.4^NS^
**cB12**^a^	**1**	–	–	–	–	–	1.4^NS^
	**2**	–	–	–	–	–	1.8^NS^
	**3**	–	–	–	–	–	5.0^NS^
**Plasma tHcy**	**1**	Vitamin B_6_ intake (mg/day)	−0.140	−0.026	−0.049, −0.004	0.021	15.1∗∗∗
		Folate intake (μg/day)	−0.047	−4.908x10^−5^	−1.426x10^−4^, 4.439x10^−5^	0.303	
		Vitamin B_12_ intake (μg/day)	−0.025	−0.001	−0.002, 0.001	0.520	
		Riboflavin intake (mg/day)	−0.009	−6.439x10^−5^	−0.001, 4.460x10^−4^	0.804	
	**2**	Plasma folate (nmol/L)	−0.214	−0.003	−0.004, −0.002	<0.001	22.7∗∗∗
		Plasma B_12_ (pmol/L)	−0.211	−1.898x10^−4^	−2.522x10^−4^, −1.273x10^−4^	<0.001	
		EGR activation coefficient	−0.064	−0.033	−0.069, 0.003	0.076	
		EAST activation coefficient	0.064	0.034	−0.003, 0.071	0.069	
	**3**	*MTHFR6*77 TT vs. CC	0.275	0.088	0.064, 0.112	<0.001	30.5∗∗∗
		Plasma B_12_ (pmol/L)	−0.208	−1.874x10^−4^	−2.476x10^−4^, −1.271x10^−4^	<0.001	
		Plasma folate (nmol/L)	−0.180	−0.003	−0.004, −0.002	<0.001	
		EAST activation coefficient	0.065	0.035	−0.001, 0.071	0.058	
		*TCII6*7 GG vs. AA	0.057	0.065	−0.011, 0.140	0.091	
		*MTRR6*6 AG vs. AA	0.057	0.014	−0.006, 0.034	0.165	
		*MTRR5*24 TT vs. CC	−0.049	−0.028	−0.054, 0.001	0.157	
		*MTR2*756 AG vs. AA	−0.047	−0.019	−0.038, 0.001	0.144	
		EGR activation coefficient	−0.036	−0.018	−0.054, 0.017	0.308	
		*MTHFR6*77 CT vs. CC	0.034	0.008	−0.010, 0.027	0.370	
		*MTRR6*6 GG vs. AA	0.031	0.009	−0.014, 0.032	0.447	
		*TCII7*76 CG vs. CC	−0.019	−0.005	−0.023, 0.014	0.620	
		*TCII6*7 AG vs. AA	0.019	0.006	−0.015, 0.027	0.584	
		*SLC19A1*80 AA vs. GG	−0.012	−0.003	−0.027, 0.020	0.774	
		*TCII7*76 GG vs. CC	−0.012	−0.004	−0.027, 0.019	0.756	
		*MTRR5*24 CT vs. CC	−0.010	−0.003	−0.020, 0.015	0.775	
		*SLC19A1*80 GA vs. GG	−0.008	−0.002	−0.022, 0.018	0.848	
		*MTR2*756 GG vs. AA	−0.007	−0.005	−0.055, 0.044	0.831	

Model 3: model 2 + polymorphism. All models were adjusted for: age, sex, BMI, socioeconomic status (low versus mid-high studies and profession), smoking status (non-smokers versus smokers), at-risk alcohol consumers ((>16 g/day in women and >24 g/day in men) versus none), creatinine (μmol/L) and energy. ∗*P* < 0.05; ∗∗*P* < 0.01; ∗∗∗*P* < 0.001.

Abbreviations. B_12_: cobalamin; B_6_: pyridoxine; BMI: body mass index; cB12: combined indicator of vitamin B_12_ status; EASTAC: erythrocyte aspartate aminotransferase activation coefficient; EGRAC: erythrocyte glutathione reductase activation coefficient; holoTC: holotranscobalamin; MTHFR: methylenetetrahydrofolate reductase; MTR: methionine transferase; MTRR: methionine transferase reductase; NS: not significant; RBCF: red blood cell folate; SLC19A1: solute carrier family 19A, member 1; TCII: transcobalamin II; tHcy: fasting total homocysteine; vs.: versus.

a Results not shown because the model was not significant.

**Fig. 1:**
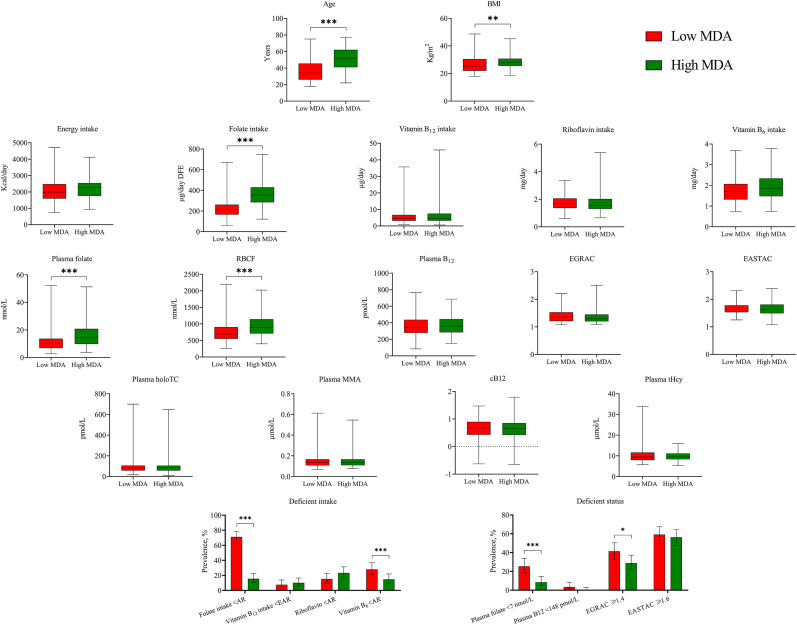
Characteristics of the study population according to Mediterranean Diet adherence. Values are reported as median (25th, 75th percentile) in the box plots and percentage (95 % CI) in the histograms. Low MDA: score ≤2.0, and high MDA: score ≥6.0. The Kruskal-Wallis test and chi-square test was used to compare differences between low MDA and high MDA. Post-hoc Bonferroni correction for multiple comparisons was applied to *P*-values. AR (European Food Safety Authority (EFSA), 2017): riboflavin: 1.3 mg/day; vitamin B_6_: women: 1.3 mg/day, and men: 1.5 mg/day, and folate: 250 μg/day of DFE. EAR (Institute of Medicine (US) Standing Committee on the Scientific Evaluation of Dietary Reference Intakes and its Panel on Folate, 1998): vitamin B_12_: 2.0 μg/day. ∗*P* < 0.05; ∗∗*P* < 0.01; ∗∗∗*P* < 0.001. Abbreviations. AR: average requirement; B_12_: cobalamin; B_6_: pyridoxine; BMI: body mass index; cB12: combined indicator of vitamin B_12_ status; DFE: dietary folate equivalents; EAR: estimated average requirement; EASTAC: erythrocyte aspartate aminotransferase activation coefficient; EGRAC: erythrocyte glutathione reductase activation coefficient; holoTC: holotranscobalamin; MDA: Mediterranean diet adherence; MMA: methylmalonic acid; RBCF: red blood cell folate; tHcy: fasting total homocysteine.

The characteristics of the study population by different age and sex groups, categorized by quartiles of Mediterranean diet adherence (MDA) are reported in Fig. 1 and Supplementary Table S3. The lowest quartile (score ≤2.0) represents the lowest MDA and the highest quartile (score ≥6.0) represents the higher MDA. Participants with the lowest MDA were younger and had lower BMI compared to those with the highest MDA, both in the overall population and in men and women ≤50 y. However, in women >50 y, those with the lowest MDA had higher BMI compared to those with the highest MDA. No differences in intakes of energy, riboflavin, vitamin B_6_ or vitamin B_12_ were observed according to MDA adherence in any sector of the population and the same was true for energy adjusted intakes of the vitamins. However, folate intake was higher in all sectors of the population with the highest MDA compared to the lowest, except in the case of women >50 y. Similar results were observed for energy-adjusted folate intake. In men ≤50 y, energy-adjusted vitamin B_6_ intake was higher in those with the best compared to the worst MDA (2.0 vs. 1.5 μg/day).

Folate, riboflavin and vitamin B_6_ intake below the AR was observed in participants in both the lowest and highest quartiles of MDA across all of the groups. Vitamin B_12_ intake below the EAR was observed in all of the groups except for men ≤50 y. There was no difference in prevalence of riboflavin intake below the AR and vitamin B_12_ intake below the EAR between the lowest and highest MDA quartiles in any of the groups. The prevalence of vitamin B_6_ intake below the AR was lower in the highest compared to the lowest MDA quartile in the overall group only. Folate intake below the AR was lower in the highest versus the lowest MDA quartiles across all of the groups. No participant exceeded the tolerable upper level (UL) of intake for vitamin B_6_ and folate.

No differences in the biomarkers EGRAC, EASTAC, plasma B_12_, holoTC, MMA, cB12 or tHcy were observed between the MDA quartiles in any of the groups. However, overall plasma folate (14.5 vs. 10.0 nmol/L) and RBCF (895.7 vs. 692.8 nmol/L), were higher in the highest versus lowest MDA quartile and this was also true for women ≤50 y.

No differences in prevalence of deficient status in EASTAC ≥1.6, RBCF <340 nmol/L, plasma B_12_ < 221 pmol/L, holoTC <35 pmol/L, MMA >0.37 μmol/L or cB12 <-0.5 were observed between the lowest versus highest MDA quartiles in any of the groups. In the overall group, prevalence of EGRAC ≥1.4, indicating riboflavin deficiency, was highest in the lowest versus highest MDA quartile, (41.4 vs. 28.9). However, this was not true for the sex and age groups. Similarly, plasma B_12_ < 148 pmol/L was most prevalent in the lowest versus highest MDA quartile (3.4 vs. 0.0 %). Plasma folate <7 nmol/L was more prevalent in the lowest versus highest MDA quartiles overall (25.4 vs. 8.5 %), in men ≤50 y (42.9 vs. 9.1 %) and in women >50 y (18.8 vs. 0.0 %). Plasma folate status <10 nmol/L, was more prevalent in the lowest quartile compared to the highest quartile (50.0 vs. 26.4 %). RBCF <906 nmol/L, was more prevalent in the lowest versus the highest MDA quartile overall (77.1 vs. 51.2 %) and in women ≤50 y (86.2 vs. 56.5 %) and men ≤50 y (82.9 vs. 57.6 %). Elevated plasma tHcy status (>15 μmol/L) was more prevalent in the lowest versus highest MDA quartile, overall (6.4 vs. 0.8 %) and in men ≤50 y (14.7 vs. 0.0 %).

Overall, only 17.4 % of the participants had good MDA. However, stratification revealed that only 8.6 % of women ≤50y, had good MDA compared to 14.0 % of men in the same age group. In contrast, in participants >50 y, 25.4 % of women and 36.2 % of men adhered well to the Mediterranean diet (Supplementary Figure S2).

**Fig. 2:**
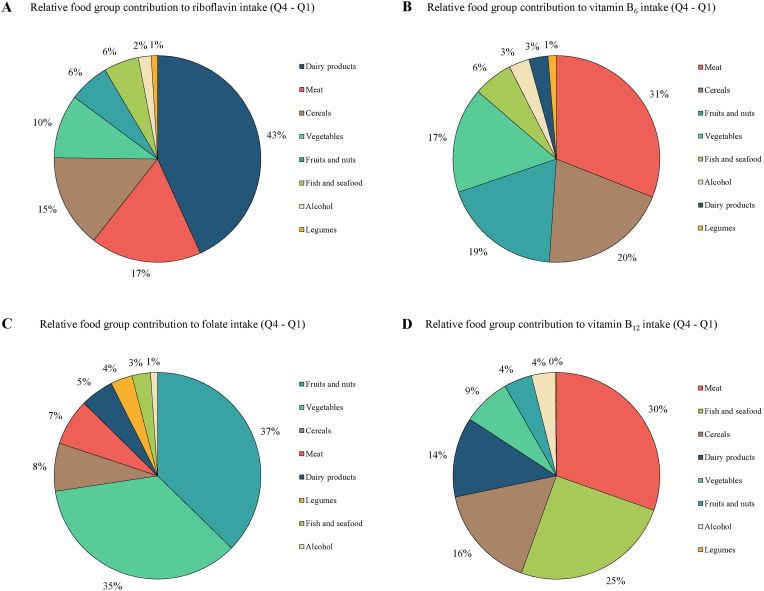
Relative contribution of food groups to differences in dietary A) riboflavin intake, B) vitamin B_6_ intake, C) folate intake, and D) vitamin B_12_ intake between the highest and lowest quartiles. The pie chart illustrates the relative contribution (%) of each food group to the total difference in mean intake of B-vitamins between the highest (Q4) and lowest (Q1) quartiles. To estimate the relative contribution of each food group to differences in dietary B-vitamin intake, participants were first categorized into quartiles (Q1 - Q4) based on their intake of each B-vitamin (riboflavin intake (A), vitamin B_6_ intake (B), folate intake (C), and vitamin B_12_ intake (D)). For each food group, the mean intake was calculated within Q1 and Q4, and the absolute difference between these two quartiles (Q4 - Q1) was computed. The sum of these differences across all food groups was then calculated, and the contribution of each food group was expressed as a percentage of this total difference. This approach reflects the proportion of the increase in B-vitamin intake from Q1 to Q4 that is attributable to each food group.

Differences in food group patterns were observed between the highest versus the lowest (reference) quartiles of intake for the different B-vitamins. Cereal, fish and seafood, vegetable, meat and alcohol consumption were all higher in the highest versus the lowest quartiles of intake for all of the B-vitamins (Supplementary Table S4). Excluding breakfast cereal consumers from the analysis did not change the results (data not shown). Among the food groups on which MDA is assessed, those contributing more than 25 % to the difference in intake between the highest versus the lowest quartiles of intake for each corresponding vitamin were as follows: dairy products for riboflavin (43 %), meat for vitamin B_6_ (31 %), fruits and nuts (37 %) and vegetables (35 %) for folate, and meat (30 %) and fish and seafood (25 %) for vitamin B_12_ (Fig. 2). The mean (±SD) intakes (g/d) of these food groups in the overall population were dairy products (297.13 ± 172.92), meat (166.28 ± 86.62), fruits and nuts (219.87 ± 169.92), vegetables (184.56 ± 104.68), and fish and seafood (75.80 ± 63.88).

**Fig. 3:**
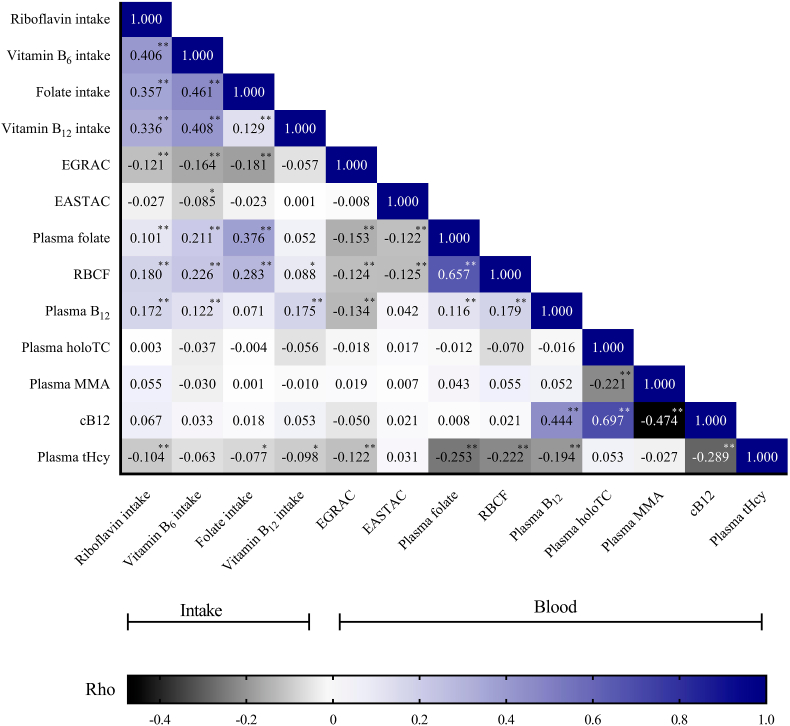
Spearman correlation matrix (rho values) of B-vitamins associations between dietary intake and blood biomarkers in 740 participants. The riboflavin, vitamin B_6_, folate and vitamin B_12_ intake were energy adjusted. Post-hoc Bonferroni correction for multiple comparisons of *P* values was applied. ∗*P* < 0.05; ∗∗*P <* 0.01. Abbreviations. B_12_: cobalamin; B_6_: pyridoxine; cB12: combined indicator of vitamin B_12_ status; EASTAC: erythrocyte aspartate aminotransferase activation coefficient; EGRAC: erythrocyte glutathione reductase activation coefficient; holoTC: holotranscobalamin; MMA: methylmalonic acid; RBCF: red blood cell folate; tHcy: fasting total homocysteine.

A Spearman correlation matrix between energy adjusted dietary intake and blood biomarkers is shown in Fig. 3. Riboflavin intake was positively associated with intake of the other B-vitamins. The strongest correlations observed between intakes were between vitamin B_6_ and folate (rho = 0.461), followed by vitamin B_6_ and vitamin B_12_. The strongest correlations between vitamin intake and biomarker status were observed between folate intake and plasma folate (rho = 0.376), followed by folate intake and RBCF (rho = 0.283), and vitamin B_6_ intake and RBCF (rho = 0.226). Riboflavin intake was associated with better riboflavin status as well as better status in both biomarkers of folate and plasma vitamin B_12_ status. Riboflavin, vitamin B_6_ and vitamin B_12_ intakes were all positively associated with plasma B_12_ concentration but none of the vitamin intakes, including, vitamin B_12_ were associated with the functional biomarkers of vitamin B_12_ status (plasma holoTC, MMA, or cB12). Regarding plasma tHcy, a functional marker of 1CM status, intake in all of the vitamins except vitamin B_6_ were weakly associated with better tHcy status. The strongest correlation with tHcy was observed in the case of riboflavin intake (rho = −0.104). Plasma folate was correlated with RBCF as well as with plasma B_12_ (rho = 0.657, rho = 0.116, respectively). Folate status (plasma and RBCF) had the strongest inverse correlations with tHcy.

**Fig. 4:**
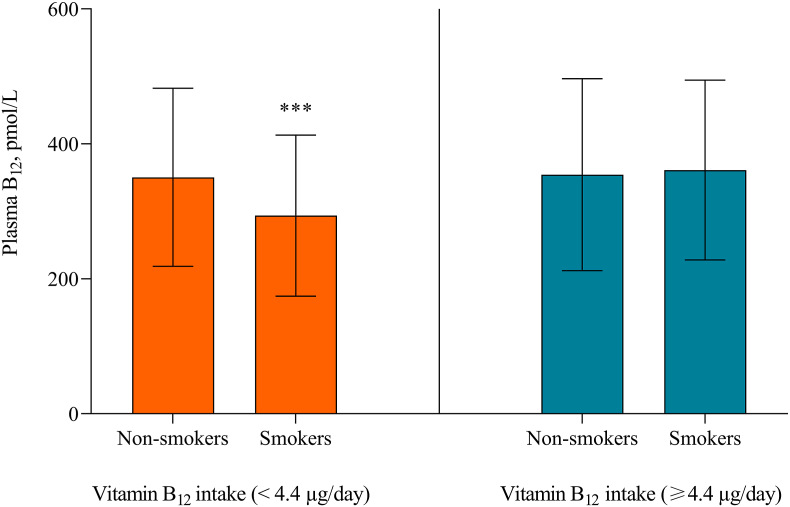
Interaction between smoking and vitamin B_12_ intake on plasma B_12_ status. Data was categorized according to vitamin B_12_ intake: below the median (<4.4 μg/day) and at or above the median (≥4.4 μg/day). Within each cobalamin intake group, plasma B_12_ status are compared between non-smokers and smokers using the Mann-Whitney *U* test. Among participants with vitamin B_12_ intake below the median, non-smokers exhibited higher median plasma B_12_ status compared to smokers (350.5 vs. 293.7 pmol/L, *P* < 0.001). In contrast, for individuals with vitamin B_12_ intake at or above the median, median plasma B_12_ status did not differ between non-smokers and smokers (354.5 vs. 361.2 pmol/L). ∗∗∗*P* < 0.001. Abbreviations. B_12_: cobalamin.

Vitamin B_12_ intake interacted with smoking status on plasma B_12_ status (*p* for interaction term = 0.031) (Fig. 4). Stratifying by median vitamin B_12_ intake (4.4 μg/d) showed that, vitamin B_12_ status was higher in non-smokers than in smokers when vitamin B_12_ intake was below the median. No difference in vitamin B_12_ status according to smoking habit occurred in participants with plasma vitamin B_12_ status above the median.

## 4 Discussion

### 4.1 Main findings of the study

To the best of our knowledge, this is the first study to assess the association between Mediterranean diet adherence and both dietary intake and biomarker status of folate, vitamin B_12_, riboflavin and vitamin B_6_ in a representative adult Km 0 Mediterranean population, free of the influence of B-vitamin supplementation and mandatory folic acid fortification. Only 17.4 % of the population adhered well to the dietary pattern for which the region is famous. In the context of the ongoing debate regarding the introduction of mandatory fortification with folic acid in European countries, dietary folate intake < AR was observed in 39.5 % of the participants. Furthermore, intakes of riboflavin and vitamin B_6_ below the AR, and vitamin B_12_ below the EAR were also observed. Plasma folate deficiency occurred in 18.6 % of the participants, and 35.9 % and 56.0 % had confirmed riboflavin and vitamin B_6_ deficiencies according to EGRAC and EASTAC status, respectively. Participants with high compared to low MDA, had higher folate intake and status. Nevertheless, 8.5 % still had plasma folate deficiency.

### 4.2 Comparison with other studies

The median folate (279.3 μg/day DFE) and vitamin B_12_ (4.4 μg/day) intakes in our study, were higher than those reported in a Mediterranean (Crete) case-control study excluding supplement or medication use affecting tHcy (folate: 254.0 μg/day DFE; vitamin B_12_: 2.2 μg/day) (Vrentzos et al., 2004). However, median plasma folate (11.1 nmol/L) was lower, plasma B_12_ (347.5 pmol/L) was higher and tHcy (9.3 μmol/L) lower, in our study compared to the Crete study (17.9 nmol/L, 240.7 pmol/L, and 12.5 μmol/L, respectively). Methodological variations as well as dietary habits may contribute to this difference (Vrentzos et al., 2004). Overall, low folate intake is common across Europe and less prevalent in the USA, where mandatory folic acid fortification and widespread supplement use have improved intake levels (Roman Viñas et al., 2011; Dhonukshe-Rutten et al., 2009). Even after mandatory fortification, intake in a portion of the U.S. population was still reported to be below the EAR for folate (Zhou et al., 2023).

Compared to a Northern Ireland study of healthy adults aged 18–92 (Jungert et al., 2020), we observed a higher prevalence of low riboflavin intake (20.1 % vs. 10.0 %) and a lower prevalence of low vitamin B_6_ intake (19.6 % vs. 29.0 %), likely due to differences in food availability, and cultural preferences.

Riboflavin status deficiency (35.9 %, based on EGRAC) aligned closely with the 37 % reported in Northern Ireland (Jungert et al., 2020). Furthermore, over half of our participants had elevated EASTAC values, indicating suboptimal vitamin B_6_ status as described in a German cohort (Waldmann et al., 2006). However, folate (18.6 %) and vitamin B_12_ (1.6 %) deficiencies differed from those reported in a cohort from Southern Spain (12.8 % and 8.6 %, respectively) (Planells et al., 2003), possibly due to differences in population age range, dietary assessment methods, and biochemical assays, although both used representative samples and excluded pregnant and lactating women. The prior study included participants aged 25–60 years and used competitive enzyme immunoassays to determine folate and vitamin B_12_ status. Conversely, our study included participants aged 18–77 years and measured folate and vitamin B_12_ status using microbiological assays. These assays measure biologically active folate and vitamin B_12_, including all active monoglutamate forms of folate and forms of vitamin B_12_ that bind intrinsic factor (excluding analogues), and are considered the gold standard. Alternatively, competitive enzyme immunoassays allow higher throughput but may present lower sensitivity, susceptibility to interference, and variable binding affinities across folate forms, as well as a limited linear range and greater analytical variability (Green et al., 2017; Molloy and Scott, 1997; Kelleher and Broin, 1991; Berger et al., 2022). The microbiological assays can be carried out on small sample volumes and are highly reproducible (Kelleher and Broin, 1991). Radio ligand and non-isotopic competitive binding assays have been shown to read higher than the microbiological assays, in the case of serum vitamin B_12_ determinations (Arnaud et al., 1994).

Low adherence to the Mediterranean diet, particularly among young participants, mirrors findings from other Spanish studies (León-Muñoz et al., 2012). A multicentre European intervention study reported the highest MDA in Sweden, followed by Italy, Germany, Belgium, Hungary, Spain, Estonia, and Cyprus (Tognon et al., 2014).

Our findings on B-vitamin intakes are consistent with European Food Safety Authority (EFSA) estimates across Europe, which report average intakes ranging from 1.4 to 2.2 mg/day for riboflavin, 1.4–3.1 mg/day for vitamin B_6_, 170–542 μg/day DFE for folate, and 4.2–8.6 μg/day for vitamin B_12_, reflecting geographic and demographic variability across Europe (EFSA Panel on Dietetic Products et al., 2017; EFSA Panel on Dietetic Products and Nutrition and Allergies (NDA), 2016; EFSA Panel on Dietetic Products and Nutrition and Allergies (NDA), 2014; EFSA Panel on Dietetic Products et al., 2015).

Dairy products were one of the main relative contributors to riboflavin intake, in line with findings from nationally representative surveys from the USA and U.K. (Institute of Medicine (US) Standing Committee on the Scientific Evaluation of Dietary Reference Intakes and its Panel on Folate, 1998; EFSA Panel on Dietetic Products et al., 2017; Drewnowski, 2011). However, dairy consumption did meet the commonly recommended maximum of three daily servings (equivalent to 600–750 g/day) (Food-Based Dietary Guidelines recommendations for milk and dairy products) and was lower than in the USA (Bowman et al., 2010). Meat was the primary source of vitamin B_6_, but meat intake exceeded the recommended levels of 0–3 servings per week (one serving = 100–125 g) (Food-Based Dietary Guidelines recommendations for meat). Lower meat consumption was reported in the NHANES (2015–2016) and the UK National Diet and Nutrition Survey (2019–2023) (Bowman et al., 2010; National Diet and Nutrition Survey). Fruits, nuts, and vegetables were the main dietary folate sources in our population, consistent with other studies from countries without mandatory folic acid fortification (Planells et al., 2003; EFSA Panel on Dietetic Products and Nutrition and Allergies (NDA), 2014). Nevertheless, our population did not meet the recommendation of a minimum of five servings of fruit and vegetables per day (equivalent to 400 g/day) (Food-Based Dietary Guidelines recommendations for fruit and vegetables), vegetable intake was higher compared to USA and UK studies (Bowman et al., 2010; National Diet and Nutrition Survey). Vitamin B_12_ intake was primarily derived from meat and fish, consistent with previous Spanish and European studies (Planells et al., 2003; EFSA Panel on Dietetic Products et al., 2015). Total fish consumption was in accordance with current dietary guidelines of at least three servings a week (equivalent to 375–450 g) (Food-Based Dietary Guidelines recommendations for fish).

### 4.3 Interpretation

Only 7.2 % of participants reported consuming commercial breakfast cereals, some of which in Spain are fortified with micronutrients including riboflavin, vitamin B_6_, folic acid and vitamin B_12_. Our findings indicate that consumption of fortified breakfast cereals contributes to higher intakes of riboflavin, vitamin B_6_, and folate, and is associated with improved plasma folate status. The reported breakfast cereal consumption was higher in a previous study of Spanish children and young adults (Van Den Boom et al., 2006). Excluding breakfast cereal consumers did not affect our results or conclusions, so they were retained in all analyses.

The absorption and bioavailability of folate, vitamin B_12_ riboflavin and vitamin B_6_, can be influenced by multiple dietary, physiological, and food preparation factors. (Baker, 1995). Natural food folate is less bioavailable compared to synthetic folic acid (McNulty and Pentieva, 2004), with bioavailability estimates around 65 % (range: 44–80 %), and is affected by various factors such as age, sex, genetic variants, and physiological conditions like pregnancy or lactation (Cuskelly et al., 1996). Folate is highly labile and easily lost during boiling or overcooking, whereas steaming or microwaving helps preserve it (Baker, 1995). Vitamin B_12_ is generally stable during cooking but may be affected by excessive heat, and its dietary sources are limited to animal products, making intake and absorption diet-dependent (Baker, 1995). Among participants with vitamin B_12_ deficiency, 33.3 % (4 participants out of 12) were over 60 years old, suggesting that malabsorption may explain the deficiency in these cases (Allen, 2010). However, despite the absence of vegans or vegetarians, the cause remains unclear for the rest. Although nine participants were being treated with proton pump inhibitors, known to lower B_12_ status, none were vitamin B_12_ deficient. Riboflavin absorption may be inhibited by minerals such as zinc, iron, and copper, as well as by caffeine, certain antibiotics, or alcohol. Riboflavin is relatively stable to heat but can be degraded by prolonged exposure to light (Baker, 1995). Vitamin B_6_ bioavailability is similarly affected by factors including thermal processing, which may induce complexes that limit absorption (Baker, 1995).

Our regression models explained a relatively low percentage of the variability in EGRAC, EASTAC, and plasma B_12_. Dietary riboflavin and vitamin B_6_ intakes were not associated with their functional biomarker status, EGRAC and EASTAC, respectively. The greatest predictor of EGRAC was plasma B_12_ and of EASTAC was plasma folate. The strongest predictor of plasma B_12_ was EGRAC. Previously, we have reported interactions between riboflavin status and genes affecting folate and vitamin B_12_ status (García-Minguillán et al., 2014). No associations were observed between polymorphisms in B_12_-related genes (MTR, MTRR, TCII) and vitamin B_12_ status. This may be due to our population-based sample, as opposed to patient samples where such genetic effects may be more evident. The low variability explained by the models suggests that additional, unexamined factors contribute to B vitamin status. Furthermore, none of the models predicting functional indicators of vitamin B_12_ status were significant despite testing the same factors that predict plasma B_12_. It is possible that vitamin B_12_ status, was not low enough to cause elevated plasma MMA. Stratifying by median plasma B_12_ status did not improve model performance for holoTC, MMA, or cB12. There is ongoing debate about the most appropriate biomarkers for assessing vitamin B_12_ status, particularly in non-clinical populations. In our study, we found no advantage of using holoTC or MMA over plasma B_12_ as indicators of vitamin B_12_ status.

Conversely, our models predicted up to 31 % of the variability in plasma folate and RBCF. In the absence of mandatory folic acid fortification, the TT genotype was more strongly associated with folate status than dietary folate intake highlighting its association with impaired folate metabolism (Frosst et al., 1995; Bueno et al., 2016; Jacques et al., 1996). The *SLC19A1* 80 AA polymorphism was also negatively associated with RBCF status (as we previously reported (Bueno et al., 2016),). With the addition of dietary data, we now observe that the association of this polymorphism with folate status is comparable in strength to that of dietary folate intake, likely due to its known role in limiting cellular folate uptake (Yates and Lucock, 2005).

The *MTHFR* 677 TT genotype was a stronger predictor of elevated tHcy status, than dietary folate intake. Plasma folate and B_12_ were negatively associated with tHcy, while EASTAC was positively associated, but not significant. There was no association between EGRAC and tHcy. The vitamin status indicator model explained tHcy variability better than the vitamin intake models. Replacing plasma B_12_ with holoTC or MMA reduced the predictive power of the model and neither was associated with tHcy.

The low MDA may be due to the change in dietary habits in Spain, driven by globalization, socio-cultural factors, and daily lifestyles that favour fast and convenience foods over traditional cooking. This shift has led to higher consumption of more protein, fat and sugar, and the loss of unique characteristics associated with the Mediterranean diet (León-Muñoz et al., 2012; Da Silva et al., 2009; Bach-Faig et al., 2011a). Furthermore, economic constraints can also limit access to key Mediterranean key foods such as fish, nuts and olive oil (that are expensive), while social changes such as the decline of family meals reduce opportunities to maintain traditional eating practices (Da Silva et al., 2009). These factors collectively impair the transgenerational uptake of the Mediterranean dietary pattern.

Even good MDA did not guarantee meeting the AR and EAR for the B-vitamins studied. A recent systematic review (Quinn et al., 2024) highlighted that countries with mandatory folic acid fortification had 50–100 % higher folate status and 25–50 % lower NTD rates compared to regions without fortification (Quinn et al., 2024). Insufficient folate supply during neural tube development in the foetus is a risk factor for grave neural tube defects leading to irreversible lifelong disabilities and/or foetal death. Before a woman knows that she's pregnant, the neural tube of the foetus has already formed, so periconceptional folic acid intake has been recommended to prevent NTD-affected pregnancies in women of reproductive age (Crider et al., 2022). This requires knowledge of the recommendation and planning of pregnancy. In Spain, where mandatory fortification is absent, a high proportion of women of reproductive age do not reach the recommended RBCF threshold (906 nmol/L) to minimize NTDs risk through diet alone (Bueno et al., 2016). These findings highlight the need to improve both MDA and B-vitamin status in the general population. Strategies could include mandatory food fortification, rather than voluntary, and enhanced promotion of traditional Mediterranean diet practices. The U.K. is set to implement the mandatory folic acid fortification (0.250 mg of folic acid, as pteroylmonoglutamic acid, per 100 g of common wheat flour) beginning December 13, 2026, with the aim of reducing NTD prevalence (The Bread and Flour).

### 4.4 Strengths and limitations

This study has several strengths. It used a randomly selected, representative sample from a Mediterranean region unexposed to mandatory B-vitamin fortification or supplement use, allowing us to examine the relationship between natural dietary B-vitamin intake and status within the context of the Mediterranean diet. Furthermore, we assessed both direct and functional biomarkers using gold-standard methods.

Limitations include potential residual confounding, despite adjusting for multiple covariates. The low predictive power of some models suggests other factors, such as the gut microbiome, may influence B-vitamin status. We have been transparent in reporting model performance and limitations, a level of detail that is often missing in previous research on this topic. Another limitation is that self-reported food records are subject to recall errors, inaccurate portion estimates, social desirability bias, and the tendency for participants to modify their eating habits while recording, often leading to underreporting of energy intake. These factors may reduce data reliability and introduce bias; however, in this study, trained dietitians carefully reviewed the records with the participants to improve accuracy. Finally, while the Trichopoulou MDA score has limitations of comparison between studies, we set out to compare B-vitamin intakes and status among quartiles of MDA adherence within our own population.

Although the data were collected between 1998 and 2002, they are relevant for consideration in policy making because they were collected free of the influence of B-vitamin use and mandatory fortification with folic acid or riboflavin. A recent study, in the same region, confirmed that 47.4 % of pregnant women did not meet the recommended red blood folate status to prevent neural tube defects (Santos-Calderón et al., 2024). The data also provide a baseline for assessing long-term trends in Mediterranean diet adherence, reflecting enduring structural determinants such as cultural food traditions and regional availability, and allow comparisons across decades to determine whether deviations from traditional diets are gradual or more recent (Bach-Faig et al., 2011b).

## 5 Conclusions

In this Mediterranean population, only 17.4 % of adults adhered well to the Mediterranean diet. Low folate intake was observed in 39.5 % of participants, and 18.6 % were classified as folate deficient. Participants with a good MDA, had better folate intake and status compared to those with lower adherence. In addition, although deficiencies in other B-vitamins were identified, their status did not differ according to MDA. These findings add to the evidence that adequate intake of natural dietary folate does not guarantee adequate folate status, and now we report this in the context of the “gold Standard” Mediterranean diet.

## CRediT authorship contribution statement

Ailende Eigbefoh-Addeh: research design, conceptualization, methodology, data curation and analysis, writing - original draft, writing - review & editing. Albert Salas-Huetos: research design, conceptualization, methodology, data curation and analysis, supervision, writing - review & editing. Carla Ramos-Rodríguez: data curation, analysis. Santiago Ceruelo: research design, clinical leadership in El Morell and La Pobla de Mafumet, participant recruitment, clinical check-ups, data collection. Lídia Ríos: clinical leadership in Cambrils, participant recruitment, clinical check-ups, data collection. Per M Ueland: Methodology. Klaus Meyer: Methodology. Joan D Fernandez-Ballart: Data curation and analysis, project administration, core methodology, funding acquisition. Michelle M Murphy: Research design, conceptualization, methodology, data curation and analysis, project administration, core methodology, funding acquisition, supervision, writing - review & editing. All authors read and approved the final manuscript.

## Funding statement

This work was supported by Institute of Salud Carlos III grants FIS 00/0954, 03/0870, 10.13039/100006301CIBER CB06/03; Agency for Administration of University and Research grant 10.13039/501100003030AGAUR 2009 10.13039/100004141SGR 1237. AE-A is a predoctoral researcher in the Unit of Preventive Medicine and Biostatistics at URV, supported by a grant from 10.13039/501100007512Universitat Rovira i Virgili and Diputació de Tarragona (2022PMF-PIPF-06). C.R.R. was a postdoctoral researcher at the 10.13039/100009674IISPV supported by a grant from 10.13039/501100003030AGAUR (INVESTIGO, 2022 INV-1 00036).

## Declaration of competing interest

The authors declare that they have no known competing financial interests or personal relationships that could have appeared to influence the work reported in this paper.
