# Supplementary material for: Prevalent low Mediterranean diet adherence and low folate status in a Spanish Km 0 Mediterranean coast population
Source: Curr Res Food Sci. 2025 Oct 7;11:101217. doi: 10.1016/j.crfs.2025.101217 (PMC12550328; doi:10.1016/j.crfs.2025.101217)
Supplement: Multimedia component 2 [file mmc2.docx]

**Figure S1.** Flow chart of participants included in the study.

Adults (aged 18 to 77 years) included in the study
N = 812

n = 788

B-vitamin users

n = 24

n = 745

Unreturned dietary records

n = 43

n = 743

Vitamin B_12_ injections

n = 3

Women
n = 388

Men
n = 352

n = 740

Impaired renal function

n = 2

**Table S1.** Differences in B-vitamin intake and status biomarkers between breakfast cereal consumers and non-consumers.

| **Variables** | **Non-consumers of breakfast cereals** | **Consumers of breakfast cereals** | ***P*** |
| --- | --- | --- | --- |
| **Dietary intake** |  |  |  |
| Energy (kcal/day)^a^ | 2135.87 (1678.95, 2610.24) | 2038.04 (1622.38, 2527.92) | 0.267 |
| Riboflavin intake (mg/day)^a^ | 1.63 (1.34, 2.02) | 2.01 (1.61, 2.44) | <0.001 |
| Vitamin B_6_ intake (mg/day)^a^ | 1.80 (1.41, 2.24) | 2.25 (1.81, 2.69) | <0.001 |
| Folate intake (µg/day DFE)^a,b^ | 272.39 (210.25, 355.48) | 351.26 (297.99, 414.10) | <0.001 |
| Vitamin B_12_ intake (µg/day)^a^ | 4.45 (3.03, 6.32) | 4.43 (3.14, 6.94) | 0.992 |
| **B-vitamin status** |  |  |  |
| EGRAC^a^ | 1.32 (1.20, 1.53) | 1.27 (1.15, 1.40) | 0.158 |
| EASTAC^a^ | 1.63 (1.52, 1.80) | 1.60 (1.51, 1.79) | 0.850 |
| Plasma folate (nmol/L)^a^ | 11.11 (7.58, 16.65) | 14.49 (9.07, 23.07) | 0.044 |
| RBCF (nmol/L)^a^ | 773.71 (602.50, 982.49) | 846.93 (595.65, 1079.37) | 0.679 |
| Plasma B_12_ (pmol/L)^a^ | 336.66 (256.92, 424.28) | 350.51 (296.26, 440.17) | 0.992 |
| Plasma holoTC (pmol/L)^a^ | 78.16 (58.21, 103.04) | 69.29 (59.49, 105.20) | 0.236 |
| Plasma MMA (µmol/L)^a^ | 0.14 (0.12, 0.16) | 0.15 (0.12, 0.19) | 0.399 |
| cB12^a^ | 0.62 (0.43, 0.85) | 0.54 (0.32, 0.92) | 0.689 |
| Plasma tHcy (µmol/L)^a^ | 9.39 (7.85, 11.13) | 8.46 (7.38, 9.71) | 0.071 |

^a^Continuous variables are presented as median (25^th^, 75^th^ percentile). ^b^µg/day of dietary folate equivalents (1 μg of DFE = 1 μg of food folate = 0.6 μg of folic acid from fortified foods and supplements) (​Institute of Medicine (US) Standing Committee on the Scientific Evaluation of Dietary Reference Intakes and its Panel on Folate, 1998​).

The Mann-Whitney test was used to compare differences between the groups.

Abbreviations. B_12_: cobalamin; B_6_: pyridoxine; cB12: combined indicator of vitamin B_12_ status; DFE: dietary folate equivalents; EASTAC: erythrocyte aspartate aminotransferase activation coefficient; EGRAC: erythrocyte glutathione reductase activation coefficient; holoTC: holotranscobalamin; MMA: methylmalonic acid; RBCF: red blood cell folate; tHcy: fasting total homocysteine.

**REFERENCES:**

Institute of Medicine (US) Standing Committee on the Scientific Evaluation of Dietary Reference Intakes and its Panel on Folate, 1998. Other B Vitamins, and Choline. Dietary Reference Intakes for Thiamin, Riboflavin, Niacin, Vitamin B6, Folate, Vitamin B12, Pantothenic Acid, Biotin, and Choline. National Academies Press (US), Washington (DC).

**Table S2.** Differences in B-vitamin intake and status biomarkers between inland vs. coastal location.

| **Variables** | **Coastal** | **Inland** | ***P*** |
| --- | --- | --- | --- |
| **Demographic information** |  |  |  |
| Age (years)^a^ | 42.0 (31.0, 56.0) | 41.0 (29.0, 52.0) | 0.423 |
| BMI (kg/m^2^)^a^ | 26.9 (23.2, 30.3) | 26.3 (23.0, 29.8) | 0.171 |
| Socioeconomic status, low studies and profession^b^ | 45.1 (40.0, 50.3) | 30.6 (26.3, 35.4) | <0.001 |
| Active smoking^b^ | 34.1 (29.3, 39.2) | 31.2 (26.7, 36.0) | 0.398 |
| Alcohol intake, prevalence of high-risk intake^b,c^ | 13.8 (10.6, 17.8) | 16.4 (13.0, 20.4) | 0.619 |
| Proton pump inhibitor use^b^ | 0.8 (0.3, 2.5) | 1.6 (0.7, 3.4) | 0.550 |
| Breakfast cereal use^b^ | 7.6 (5.3, 10.9) | 6.8 (4.6, 9.7) | 0.646 |
| **Dietary intake** |  |  |  |
| Energy (kcal/day)^a^ | 2084.1 (1614.2, 2606.1) | 2174.1 (1722.9, 2609.4) | 0.185 |
| Riboflavin intake (mg/day)^a^ | 1.6 (1.3, 2.1) | 1.7 (1.4, 2) | 0.883 |
| Vitamin B_6_ intake (mg/day)^a^ | 1.9 (1.5, 2.3) | 1.8 (1.4, 2.2) | 0.040 |
| Folate intake (µg/day DFE)^a,d^ | 284.5 (225.3, 372.5) | 271.9 (207.1, 352.5) | 0.185 |
| Vitamin B_12_ intake (µg/day)^a^ | 4.4 (2.8, 6.1) | 4.5 (3.2, 6.8) | 0.769 |
| **B-vitamin status** |  |  |  |
| EGRAC^a^ | 1.2 (1.2, 1.4) | 1.4 (1.3, 1.6) | <0.001 |
| EASTAC^a^ | 1.6 (1.5, 1.7) | 1.7 (1.5, 1.8) | 0.003 |
| Plasma folate (nmol/L)^a^ | 10.8 (7.2, 16.3) | 11.6 (8.5, 16.1) | 0.502 |
| RBCF (nmol/L)^a^ | 737.7 (606.6, 969.7) | 851.8 (630.2, 1067.8) | <0.001 |
| Plasma B_12_ (pmol/L)^a^ | 342.7 (262.7, 440) | 352.7 (285.5, 438.9) | 0.173 |
| Plasma holoTC (pmol/L)^a^ | 80.2 (59.8, 106.3) | 78.1 (56.8, 105.3) | 0.657 |
| Plasma MMA (µmol/L)^a^ | 0.1 (0.1, 0.2) | 0.1 (0.1, 0.2) | 0.407 |
| cB12^a^ | 0.6 (0.4, 0.8) | 0.7 (0.4, 0.9) | 0.631 |
| Plasma tHcy (µmol/L)^a^ | 10.1 (8.6, 11.9) | 8.7 (7.3, 10.6) | <0.001 |

^a^Continuous variables are reported as median (25^th^, 75^th^ percentile). ^b^Percentage (95% confidence interval). ^c^Prevalence of high-risk intake > 24 g/day in men and > 16 g/day in women. ^d^µg/day of dietary folate equivalents (1 μg of DFE = 1 μg of food folate = 0.6 μg of folic acid from fortified foods and supplements) (​Institute of Medicine (US) Standing Committee on the Scientific Evaluation of Dietary Reference Intakes and its Panel on Folate, 1998​).

The Mann-Whitney test was used to compare differences between the groups.

Abbreviations. B_12_: cobalamin; B_6_: pyridoxine; cB12: combined indicator of vitamin B_12_ status; DFE: dietary folate equivalents; EASTAC: erythrocyte aspartate aminotransferase activation coefficient; EGRAC: erythrocyte glutathione reductase activation coefficient; holoTC: holotranscobalamin; MMA: methylmalonic acid; RBCF: red blood cell folate; tHcy: fasting total homocysteine.

**REFERENCES:**

Institute of Medicine (US) Standing Committee on the Scientific Evaluation of Dietary Reference Intakes and its Panel on Folate, 1998. Other B Vitamins, and Choline. Dietary Reference Intakes for Thiamin, Riboflavin, Niacin, Vitamin B6, Folate, Vitamin B12, Pantothenic Acid, Biotin, and Choline. National Academies Press (US), Washington (DC).

**Table S3.** Characteristics of the study population according to quartiles of Mediterranean diet adherence.

|  | **Women ≤ 50 y**  **n = 266** | | **Men ≤ 50 y**  **n = 236** | | **Women > 50 y**  **n = 122** | | **Men > 50 y**  **n = 116** | |
| --- | --- | --- | --- | --- | --- | --- | --- | --- |
|  | **1^st^ Q  n = 58** | **4^th^ Q  n = 23** | **1^st^ Q  n = 35** | **4^th^ Q  n = 33** | **1^st^ Q  n = 16** | **4^th^ Q  n = 31** | **1^st^ Q  n = 9** | **4^th^ Q  n = 42** |
| Age (years)^a^ | 31.0 (25.8, 39.3) | 36.0 (27.0, 43.0) | 29.0 (23.0, 34.0) | 39.0 (31.0, 44.0)^***^ | 62.0 (53.3, 69.3) | 62.0 (56.0, 71.0) | 63.0 (57.0, 69.0) | 57.5 (55.0, 67.0) |
| BMI (kg/m^2^)^a^ | 23.9 (20.8, 28.1) | 23.3 (21.1, 29.8) | 24.4 (21.8, 27.7) | 27.2 (25.7, 30.2)^*^ | 33.8 (32.0, 38.3) | 29.3 (27.1, 31.0)^**^ | 26.7 (23.6, 35.1) | 28.9 (27.5, 32.4) |
| **Dietary intake** | | | | | | | | |
| Energy (kcal/day)^a^ | 1890.0 (1550.6, 2155.9) | 1967.9 (1683.4, 2288.3) | 2701.5 (2364.3, 3160.8) | 2545.2 (2332.7, 2992.2) | 1468.1 (1209.2, 1675.2) | 1648.7 (1526.8, 1940.1) | 2050.1 (1747.0, 2619.5) | 2359.7 (1995.9, 2613.8) |
| Riboflavin intake (mg/day)^a^ | 1.6 (1.4, 1.9) | 1.5 (1.2, 1.7) | 2.0 (1.7, 2.5) | 1.8 (1.5, 2.3) | 1.6 (1.3, 1.7) | 1.3 (1.1, 1.7) | 1.7 (1.3, 2.2) | 1.6 (1.4, 2.2) |
| Energy adjusted riboflavin intake^a^ | 1.8 (1.7, 1.9) | 1.8 (1.6, 1.9) | 1.9 (1.8, 2.0) | 1.8 (1.7, 2.0) | 1.8 (1.7, 2.0) | 1.7 (1.6, 1.8) | 1.8 (1.7, 2.0) | 1.8 (1.7, 1.9) |
| Vitamin B_6_ intake (mg/day)^a^ | 1.5 (1.2, 1.9) | 1.6 (1.4, 1.9) | 2.0 (1.6, 2.2) | 2.4 (2.0, 2.8) | 1.5 (1.2, 2.0) | 1.4 (1.2, 1.5) | 2.1 (1.7, 2.5) | 2.0 (1.6, 2.5) |
| Energy adjusted vitamin B_6_ intake^a^ | 1.7 (1.5, 2.0) | 1.8 (1.7, 2.0) | 1.5 (1.3, 2.0) | 2.0 (1.8, 2.3)^*^ | 1.9 (1.7, 2.3) | 1.8 (1.5, 2.0) | 2.2 (1.8, 2.5) | 2.0 (1.5, 2.4) |
| Folate intake (µg/day DFE)^a,c^ | 200.3 (149.2, 246.3) | 329.5 (262.5, 390.3)^**^ | 223.1 (193.4, 278.7) | 368.2 (316.6, 482.3)^***^ | 207.9 (166.6, 296.7) | 293.0 (248.9, 395.6) | 227.9 (183.9, 278.8) | 385.7 (314.8, 483.8)^**^ |
| Energy adjusted folate intake^a^ | 223.8 (183.7, 273.1) | 354.6 (277.1, 415.0)^***^ | 184.4 (143.1, 249.9) | 345.3 (295.1, 432.5)^***^ | 252.0 (191.3, 358.9) | 325.7 (277.7, 408.4) | 221.1 (189.5, 260.9) | 383.2 (299.7, 466.0)^**^ |
| Vitamin B_12_ intake (µg/day)^a^ | 4.1 (3.0, 6.3) | 3.7 (2.4, 5.2) | 5.3 (4.5, 7.3) | 5.0 (3.7, 7.7) | 3.6 (2.2, 6.6) | 3.7 (1.9, 7.4) | 4.7 (3.8, 6.9) | 5.3 (3.4, 8.4) |
| Energy adjusted vitamin B_12_ intake^a^ | 4.7 (3.7, 6.6) | 4.3 (2.9, 5.4) | 4.3 (3.5, 5.8) | 4.6 (3.2, 6.9) | 4.5 (3.8, 7.9) | 4.0 (2.8, 7.4) | 4.7 (3.6, 6.3) | 5.4 (2.7, 8.4) |
| **Low and high dietary intake** | | | | | | | | |
| Riboflavin intake <PRI^b,d^ | 43.1 (31.2, 55.9) | 65.2 (44.9, 81.2) | 17.1 (8.1, 32.7) | 36.4 (22.2, 53.4) | 56.3 (33.2, 76.9) | 71.0 (53.4, 83.9) | 44.4 (18.9, 73.3) | 50.0 (35.5, 64.5) |
| Riboflavin intake <AR^b,e^ | 20.7 (12.3, 32.8) | 26.1 (12.5, 46.5) | 2.9 (0.5, 14.5) | 9.1 (3.1, 23.6) | 31.3 (14.2, 55.6) | 45.2 (29.2, 62.2) | 0.0 (0.0, 29.9) | 16.7 (8.3, 30.6) |
| Riboflavin intake >UL^b,f^ | - | - | - | - | - | - | - | - |
| Vitamin B_6_ intake <PRI^b,d^ | 53.4 (40.8, 65.7) | 47.8 (29.2, 67.0) | 31.4 (18.6, 48.0) | 15.2 (6.7, 30.9) | 50.0 (28.0, 72.0) | 77.4 (60.2, 88.6) | 22.2 (6.3, 54.7) | 31.0 (19.1, 46.0) |
| Vitamin B_6_ intake <AR^b,e^ | 32.8 (22.1, 45.6) | 17.4 (7.0, 37.1) | 20.0 (10.0, 35.9) | 6.1 (1.7, 19.6) | 37.5 (18.5, 61.4) | 35.5 (21.1, 53.1) | 11.1 (2.0, 43.5) | 4.8 (1.3, 15.8) |
| Vitamin B_6_ intake >UL^b,f^ | 0.0 (0.0, 6.2) | 0.0 (0.0, 14.3) | 0.0 (0.0, 9.9) | 0.0 (0.0, 10.4) | 0.0 (0.0, 19.4) | 0.0 (0.0, 10.7) | 0.0 (0.0, 29.9) | 0.0 (0.0, 8.4) |
| Folate intake <PRI^b,d^ | 91.4 (81.4, 96.3) | 52.2 (33.0, 70.8)^***^ | 85.7 (70.6, 93.7) | 33.3 (19.8, 50.4)^***^ | 81.3 (57.0, 93.4) | 58.1 (40.8, 73.6) | 100.0 (70.1, 100.0) | 28.6 (17.2, 43.6)^***^ |
| Folate intake <AR^b,e^ | 75.9 (63.5, 85.0) | 21.7 (9.7, 41.9)^***^ | 65.7 (49.2, 79.2) | 9.1 (3.1, 23.6)^***^ | 75.0 (50.5, 89.8) | 29.0 (16.1, 46.6)^**^ | 55.6 (26.7, 81.1) | 7.1 (2.5, 19.0)^***^ |
| Folate intake >UL^b,f^ | 0.0 (0.0, 6.2) | 0.0 (0.0, 14.3) | 0.0 (0.0, 9.9) | 0.0 (0.0, 10.4) | 0.0 (0.0, 19.4) | 0.0 (0.0, 10.7) | 0.0 (0.0, 29.9) | 0.0 (0.0, 8.4) |
| Vitamin B_12_ intake <RDA^b,g^ | 12.1 (6.0, 22.9) | 26.1 (12.5, 46.5) | 0.0 (0.0, 9.9) | 6.1 (1.7, 19.6) | 25.0 (10.2, 49.5) | 38.7 (23.7, 56.2) | 11.1 (2.0, 43.5) | 7.1 (2.5, 19.0) |
| Vitamin B_12_ intake <EAR^b,h^ | 8.6 (3.7, 18.6) | 17.4 (7.0, 37.1) | 0.0 (0.0, 9.9) | 0.0 (0.0, 10.4) | 18.8 (6.6, 43.0) | 25.8 (13.7, 43.2) | 11.1 (2.0, 43.5) | 2.4 (0.4, 12.3) |
| Vitamin B_12_ intake >UL^b,f^ | - | - | - | - | - | - | - | - |
| **B-vitamin status** | | | | | | | | |
| EGRAC^a^ | 1.3 (1.2, 1.6) | 1.3 (1.3, 1.6) | 1.4 (1.3, 1.6) | 1.3 (1.2, 1.5) | 1.2 (1.2, 1.4) | 1.3 (1.2, 1.4) | 1.3 (1.1, 1.4) | 1.3 (1.2, 1.4) |
| EASTAC^a^ | 1.7 (1.6, 1.8) | 1.6 (1.5, 1.8) | 1.6 (1.5, 1.8) | 1.7 (1.5, 1.8) | 1.5 (1.5, 1.8) | 1.6 (1.5, 1.9) | 1.6 (1.3, 1.6) | 1.6 (1.4, 1.8) |
| Plasma folate (nmol/L)^a^ | 9.3 (7.3, 12.3) | 14.3 (10.0, 18.5)^*^ | 9.1 (5.2, 11.8) | 10.7 (8.7, 16.7) | 15.2 (8.2, 22.1) | 15.8 (11.0, 24.0) | 13.6 (11.3, 17.6) | 15.7 (10.5, 23.1) |
| RBCF (nmol/L)^a^ | 628.0 (531.3, 807.2) | 810.3 (667.7, 1228.2)^*^ | 685.3 (537.3, 868.4) | 842.1 (647.0, 1021.5) | 1001.1 (798.1, 1381.1) | 936.6 (759.5, 1179.2) | 887.5 (712.4, 1035.5) | 904.6 (721.5, 1191.0) |
| Plasma B_12_ (pmol/L)^a^ | 316.3 (277.3, 422.4) | 380.2 (267.3, 440.9) | 381.4 (257.3, 478.4) | 359.3 (305.1, 447.7) | 400.0 (312.6, 499.6) | 388.2 (343.5, 441.7) | 362.6 (268.0, 475.2) | 320.0 (249.7, 447.4) |
| Plasma holoTC (pmol/L)^a^ | 84.9 (57.7, 110.5) | 85.4 (49.6, 100.8) | 84.2 (64.0, 108.6) | 71.2 (54.0, 91.5) | 67.0 (48.5, 98.5) | 90.7 (62.5, 115.8) | 80.5 (67.2, 87.2) | 84.6 (62.8, 115.2) |
| Plasma MMA (µmol/L)^a^ | 0.1 (0.1, 0.2) | 0.1 (0.1, 0.2) | 0.1 (0.1, 0.2) | 0.2 (0.1, 0.2) | 0.1 (0.1, 0.2) | 0.1 (0.1, 0.2) | 0.1 (0.1, 0.2) | 0.1 (0.1, 0.2) |
| cB12^a^ | 0.6 (0.4, 0.9) | 0.7 (0.5, 0.9) | 0.8 (0.4, 0.9) | 0.5 (0.3, 0.7) | 0.6 (0.3, 1.0) | 0.7 (0.5, 1.0) | 0.6 (0.4, 0.7) | 0.6 (0.5, 0.9) |
| Plasma tHcy (µmol/L)^a^ | 8.9 (7.2, 10.6) | 8.1 (7.0, 8.8) | 10.2 (8.6, 12.0) | 9.3 (8.4, 11.0) | 10.0 (7.6, 11.8) | 9.8 (8.4, 11.1) | 10.1 (8.1, 12.3) | 10.7 (9.2, 12.1) |
| **Low B-vitamin status** | | | | | | | | |
| EGRAC ≥1.4^b^ | 41.1 (29.2, 54.1) | 43.5 (25.6, 63.2) | 54.3 (38.2, 69.5) | 34.4 (20.4, 51.7) | 25.0 (10.2, 49.5) | 22.6 (11.4, 39.8) | 22.2 (6.3, 54.7) | 21.4 (11.7, 35.9) |
| EASTAC ≥1.6^b^ | 67.9 (54.8, 78.6) | 60.9 (40.8, 77.8) | 62.9 (46.3, 76.8) | 59.4 (42.3, 74.5) | 40.0 (19.8, 64.3) | 64.5 (46.9, 78.9) | 22.2 (6.3, 54.7) | 45.2 (31.2, 60.1) |
| Plasma folate <7 nmol/L^b^ | 20.7 (12.3, 32.8) | 17.4 (7.0, 37.1) | 42.9 (28.0, 59.1) | 9.1 (3.1, 23.6)^**^ | 18.8 (6.6, 43.0) | 0.0 (0.0, 11.0)^*^ | 0.0 (0.0, 29.9) | 9.5 (3.8, 22.1) |
| Plasma folate <10 nmol/L^b^ | 53.4 (40.8, 65.7) | 26.1 (12.5, 46.5)^*^ | 62.9 (46.3, 76.8) | 48.5 (32.5, 64.8) | 37.5 (18.5, 61.4) | 12.9 (5.1, 28.9)^*^ | 0.0 (0.0, 29.9) | 19.0 (10.0, 33.3) |
| RBCF <340 nmol/L^b^ | 3.4 (1.0, 11.7) | 0.0 (0.0, 14.3) | 2.9 (0.5, 14.5) | 0.0 (0.0, 10.4) | 0.0 (0.0, 19.4) | 0.0 (0.0, 11.0) | 0.0 (0.0, 29.9) | 0.0 (0.0, 8.4) |
| RBCF <906 nmol/L^b^ | 86.2 (75.1, 92.8) | 56.5 (36.8, 74.4)^**^ | 82.9 (67.3, 91.9) | 57.6 (40.8, 72.8)^*^ | 37.5 (18.5, 61.4) | 41.9 (26.4, 59.2) | 66.7 (35.4, 87.9) | 50.0 (35.5, 64.5) |
| Plasma B_12_ <148 pmol/L^b^ | 3.4 (1.0, 11.7) | 0.0 (0.0, 14.3) | 2.9 (0.5, 14.5) | 0.0 (0.0, 10.4) | 6.3 (1.1, 28.3) | 0.0 (0.0, 11.0) | 0.0 (0.0, 29.9) | 0.0 (0.0, 8.4) |
| Plasma B_12_ <221 pmol/L^b^ | 10.3 (4.8, 20.8) | 8.7 (2.4, 26.8) | 17.1 (8.1, 32.7) | 6.1 (1.7, 19.6) | 12.5 (3.5, 36.0) | 3.2 (0.6, 16.2) | 0.0 (0.0, 29.9) | 11.9 (5.2, 25.0) |
| Plasma holoTC <35 pmol/L^b^ | 3.6 (1.0, 12.3) | 0.0 (0.0, 14.9) | 5.7 (1.6, 18.6) | 9.4 (3.2, 24.2) | 0.0 (0.0, 19.4) | 6.5 (1.8, 20.7) | 0.0 (0.0, 29.9) | 0.0 (0.0, 8.4) |
| Plasma MMA >0.37 µmol/L^b^ | 1.7 (0.3, 9.1) | 0.0 (0.0, 14.3) | 2.9 (0.5, 14.5) | 6.1 (1.7, 19.6) | 0.0 (0.0, 19.4) | 3.2 (0.6, 16.2) | 0.0 (0.0, 29.9) | 0.0 (0.0, 8.4) |
| cB12 <-0.5^b^ | 0.0 (0.0, 6.2) | 0.0 (0.0, 14.3) | 2.9 (0.5, 14.5) | 3.0 (0.5, 15.3) | 6.3 (1.1, 28.3) | 3.2 (0.6, 16.2) | 0.0 (0.0, 29.9) | 0.0 (0.0, 8.4) |
| Plasma tHcy >15 µmol/L^b^ | 1.9 (0.3, 9.9) | 0.0 (0.0, 14.3) | 14.7 (6.4, 30.1) | 0.0 (0.0, 11.4)^*^ | 7.1 (1.3, 31.5) | 0.0 (0.0, 11.7) | 0.0 (0.0, 29.9) | 2.6 (0.5, 13.5) |

^a^Median (25^th^, 75^th^ percentile). ^b^Percentage (95% confidence interval). ^c^µg/day of dietary folate equivalents (1 μg of DFE = 1 μg of food folate = 0.6 μg of folic acid from fortified foods and supplements) (​Institute of Medicine (US) Standing Committee on the Scientific Evaluation of Dietary Reference Intakes and its Panel on Folate, 1998​). ^d^Population reference intake (PRI) (​European Food Safety Authority (EFSA), 2017​): riboflavin: 1.6 mg/day; vitamin B_6_: women: 1.6 mg/day, and men: 1.7 mg/day, and folate: 330 µg/day of DFE. ^e^Average requirement (AR) (​European Food Safety Authority (EFSA), 2017​): riboflavin: 1.3 mg/day; vitamin B_6_: women: 1.3 mg/day, and men: 1.5 mg/day, and folate: 250 µg/day of DFE. ^f^Upper level (UL) (​European Food Safety Authority (EFSA), 2024​): riboflavin: no adequate data to derive a UL; vitamin B_6_: 25 mg/day; folate: 1000 µg/day of DFE; vitamin B_12_: no defined adverse effects. ^g^Recommended dietary allowance (RDA) (​Institute of Medicine (US) Standing Committee on the Scientific Evaluation of Dietary Reference Intakes and its Panel on Folate, 1998​): vitamin B_12_: 2.4 µg/day. ^h^Estimated average requirement (EAR) (​Institute of Medicine (US) Standing Committee on the Scientific Evaluation of Dietary Reference Intakes and its Panel on Folate, 1998​): vitamin B_12_: 2.0 µg/day.

The Kruskal-Wallis test and chi-square test were used to compare differences within quartiles. Post-hoc Bonferroni correction of *P*-values was performed to correct for multiple comparisons. 1^st^ Q (low MDA): score ≤ 2.0, 4^th^ Q (high MDA): score ≥ 6.0. ^*^*P* < 0.05; ^**^*P* < 0.01; ^***^*P* < 0.001.

Abbreviations. AR: average requirement; B12: cobalamin; B6: pyridoxine; BMI: body mass index; cB12: combined indicator of vitamin B_12_ status; DFE: dietary folate equivalents; EAR: estimated average requirement; EASTAC: erythrocyte aspartate aminotransferase activation coefficient; EGRAC: erythrocyte glutathione reductase activation coefficient; holoTC: holotranscobalamin; MDA: Mediterranean diet adherence; MMA: methylmalonic acid; PRI: population reference intake; Q: quartile; RBCF: red blood cell folate; RDA: recommended dietary allowance; tHcy: fasting total homocysteine; UL: upper intake level; y: year.

**REFERENCES:**

European Food Safety Authority (EFSA), 2017. Dietary reference values for nutrients summary report. EFSA J. 14. doi:10.2903/sp.efsa.2017.e15121.

European Food Safety Authority (EFSA), 2024. Overview on tolerable upper intake levels as derived by the Scientific committee on food (SCF) and the EFSA panel on dietetic products, nutrition and allergies (NDA). EFSA J. Available at: https://www.efsa.europa.eu/sites/default/files/2024-05/ul-summary-report.pdf.

Institute of Medicine (US) Standing Committee on the Scientific Evaluation of Dietary Reference Intakes and its Panel on Folate, 1998. Other B Vitamins, and Choline. Dietary Reference Intakes for Thiamin, Riboflavin, Niacin, Vitamin B6, Folate, Vitamin B12, Pantothenic Acid, Biotin, and Choline. National Academies Press (US), Washington (DC).

**Figure S2.** Percentage of participants with good Mediterranean diet adherence.

**
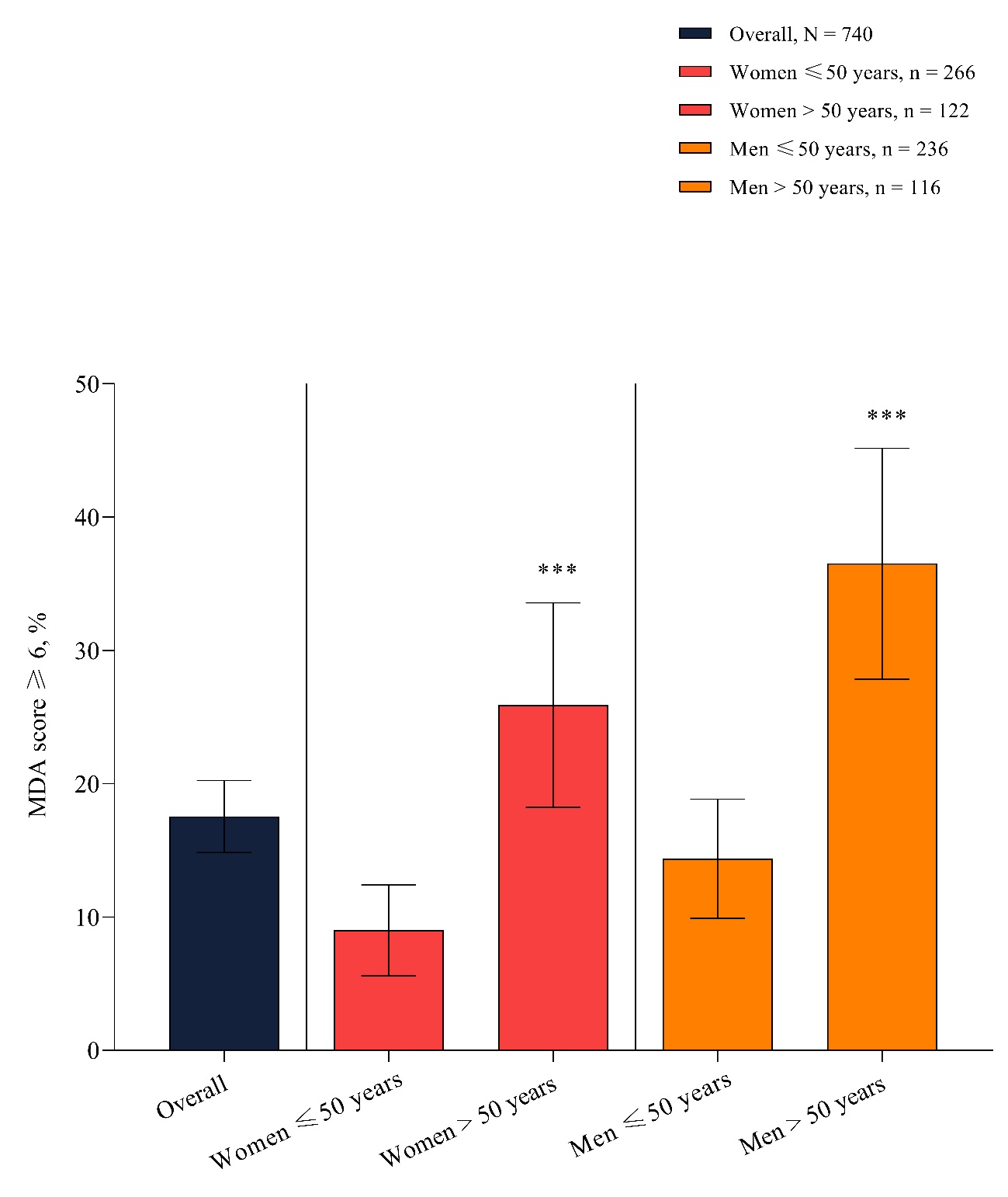
**

High MDA was defined as score ≥ 6.0 (Trichopoulou et al., 2003). The sample comprised: 266 women ≤50 years, 236 men ≤50 years, 122 women >50 years, and 116 men >50 years. Older participants (>50 years) showed better MDA compared to younger counterparts (*P* < 0.001). ^***^*P* < 0.001.

Abbreviations. MDA: Mediterranean diet adherence.

**REFERENCES:**

Trichopoulou, A., Costacou, T., Bamia, C., Trichopoulos, D., 2003. Adherence to a mediterranean diet and survival in a Greek population. N. Engl. J. Med. 348, 2599–2608. doi:10.1056/NEJMoa025039.

**Table S4.** Food group consumption according to quartiles of A) riboflavin intake, B) vitamin B_6_ intake, C) folate intake, and D) vitamin B_12_ intake.

| **A) Riboflavin intake** | **Variables** | **1^st^ Quartile**  **n=186** | **2^nd^ Quartile**  **n=186** | **3^rd^ Quartile**  **n=188** | **4^th^ Quartile**  **n=184** | ***P*** |
| --- | --- | --- | --- | --- | --- | --- |
|  | MDA score*; median [min-max] | 4 [0-8] ^a^ | 4 [0-8] ^a^ | 4 [0-8] ^b^ | 4 [1-8] ^a^ | 0.032 |
|  | Vegetables (g/d); mean (±SD) | 162.40 (98.45) ^a^ | 179.41 (103.66) ^a^ | 185.13 (93.44) ^a^ | 218.24 (119.81) ^b^ | <0.001 |
|  | Legumes (g/d); mean (±SD) | 12.84 (19.90) ^a^ | 16.25 (20.43) ^a^ | 16.73 (22.15) ^a^ | 16.97 (27.22) ^a^ | 0.256 |
|  | Fruits and nuts (g/d); mean (±SD) | 207.45 (175.77) ^a^ | 210.23 (142.11) ^a^ | 226.65 (173.43) ^a^ | 249.79 (202.93) ^a^ | 0.077 |
|  | Dairy products (g/d); mean (±SD) | 178.12 (108.48) ^a^ | 287.52 (127.34) ^b^ | 353.85 (148.77) ^c^ | 393.48 (204.74) ^c^ | <0.001 |
|  | Cereals (g/d); mean (±SD) | 151.45 (79.00) ^a^ | 172.89 (74.78) ^a,b^ | 194.19 (94.89) ^b^ | 223.79 (99.54) ^c^ | <0.001 |
|  | Meat (g/d); mean (±SD) | 127.36 (67.18) ^a^ | 145.45 (72.21) ^a^ | 177.42 (85.58) ^b^ | 210.26 (96.47) ^c^ | <0.001 |
|  | Fish and seafood (g/d); mean (±SD) | 64.61 (56.58) ^a^ | 71.33 (51.20) ^a^ | 75.24 (69.09) ^a,b^ | 89.35 (71.56) ^b,c^ | 0.002 |
|  | MUFA/SFA (ratio/d); mean (±SD) | 2.04 (0.62) ^a^ | 1.94 (0.53) ^a,b^ | 1.82 (0.50) ^b^ | 1.83 (0.47) ^b^ | <0.001 |
|  | Alcohol (g/d); mean (±SD) | 7.43 (16.30) ^a^ | 7.50 (13.69) ^a^ | 10.50 (19.01) ^a^ | 16.37 (23.69) ^b^ | <0.001 |
| **B) Vitamin B_6_ intake** | **Variables** | **1^st^ Quartile**  **n=187** | **2^nd^ Quartile**  **n=184** | **3^rd^ Quartile**  **n=188** | **4^th^ Quartile**  **n=185** | ***P*** |
|  | MDA score*; median [min-max] | 4 [0-8] ^a^ | 4 [0-7] ^a^ | 4 [0-8] ^a,b^ | 4 [1-8] ^a,c^ | 0.005 |
|  | Vegetables (g/d); mean (±SD) | 146.96 (97.54) ^a^ | 180.86 (85.81) ^b^ | 188.19 (100.49) ^b,c^ | 229.17 (121.20) ^d^ | <0.001 |
|  | Legumes (g/d); mean (±SD) | 12.74 (21.37) ^a^ | 15.60 (18.85) ^a^ | 16.25 (22.01) ^a^ | 18.23 (27.22) ^a^ | 0.130 |
|  | Fruits and nuts (g/d); mean (±SD) | 188.98 (159.47) ^a^ | 214.41 (154.00) ^a^ | 206.26 (151.01) ^a^ | 284.82 (214.92) ^b^ | <0.001 |
|  | Dairy products (g/d); mean (±SD) | 284.66 (161.82) ^a^ | 305.42 (148.24) ^a^ | 308.58 (171.02) ^a^ | 314.01 (201.49) ^a^ | 0.373 |
|  | Cereals (g/d); mean (±SD) | 141.51 (71.84) ^a^ | 165.59 (72.89) ^b^ | 199.94 (92.84) ^c^ | 235.10 (97.30) ^d^ | <0.001 |
|  | Meat (g/d); mean (±SD) | 100.78 (48.61) ^a^ | 136.85 (55.89) ^b^ | 183.37 (69.51) ^c^ | 239.39 (95.95) ^d^ | <0.001 |
|  | Fish and seafood (g/d); mean (±SD) | 61.19 (50.18) ^a^ | 75.24 (62.98) ^a,b^ | 75.48 (58.65) ^a,b^ | 88.61 (75.90) ^b^ | <0.001 |
|  | MUFA/SFA (ratio/d); mean (±SD) | 1.91 (0.62) ^a^ | 1.95 (0.54) ^a^ | 1.86 (0.55) ^a^ | 1.91 (0.43) ^a^ | 0.468 |
|  | Alcohol (g/d); mean (±SD) | 5.23 (14.61) ^a^ | 7.41 (12.12) ^a,b^ | 10.54 (16.52) ^b,c^ | 18.60 (26.28) ^c^ | <0.001 |
| **C) Folate intake** | **Variables** | **1^st^ Quartile**  **n=186** | **2^nd^ Quartile**  **n=186** | **3^rd^ Quartile**  **n=186** | **4^th^ Quartile**  **n=186** | ***P*** |
|  | MDA score*; median [min-max] | 3 [0-7] ^a^ | 4 [1-7] ^b^ | 4 [2-8] ^c^ | 5 [1-8] ^c^ | <0.001 |
|  | Vegetables (g/d); mean (±SD) | 110.65 (63.57) ^a^ | 151.65 (73.54) ^b^ | 207.34 (88.32) ^c^ | 275.18 (111.82) ^d^ | <0.001 |
|  | Legumes (g/d); mean (±SD) | 8.96 (16.22) ^a^ | 13.05 (17.42) ^a,b^ | 17.31 (21.18) ^b,c^ | 23.47 (30.36) ^d^ | <0.001 |
|  | Fruits and nuts (g/d); mean (±SD) | 143.84 (141.26) ^a^ | 199.30 (141.91) ^b^ | 232.01 (139.54) ^b^ | 318.71 (218.28) ^c^ | <0.001 |
|  | Dairy products (g/d); mean (±SD) | 288.62 (160.14) ^a^ | 306.83 (171.19) ^a^ | 290.67 (153.41) ^a^ | 326.43 (197.72) ^a^ | 0.121 |
|  | Cereals (g/d); mean (±SD) | 167.77 (73.78) ^a^ | 178.42 (92.10) ^a^ | 189.50 (91.27) ^a,b^ | 206.30 (102.57) ^b^ | <0.001 |
|  | Meat (g/d); mean (±SD) | 145.48 (75.77) ^a^ | 166.48 (89.71) ^a,b^ | 169.96 (96.80) ^b,c^ | 178.22 (81.21) ^b,c^ | 0.002 |
|  | Fish and seafood (g/d); mean (±SD) | 64.09 (52.83) ^a^ | 70.43 (57.45) ^a^ | 88.18 (71.08) ^b^ | 77.67 (67.63) ^a,b^ | 0.002 |
|  | MUFA/SFA (ratio/d); mean (±SD) | 1.74 (0.48) ^a^ | 1.85 (0.56) ^a^ | 2.00 (0.56) ^b^ | 2.04 (0.53) ^b^ | <0.001 |
|  | Alcohol (g/d); mean (±SD) | 6.41 (15.88) ^a^ | 10.85 (17.88) ^a,b^ | 13.37 (20.57) ^b^ | 11.21 (20.16) ^a,b^ | 0.004 |
| **D) Vitamin B_12_ intake** | **Variables** | **1^st^ Quartile**  **n=187** | **2^nd^ Quartile**  **n=185** | **3^rd^ Quartile**  **n=186** | **4^th^ Quartile**  **n=186** | ***P*** |
|  | MDA score*; median [min-max] | 4 [0-8] ^a^ | 4 [0-8] ^a^ | 4 [0-8] ^a^ | 4 [1-8] ^a^ | 0.194 |
|  | Vegetables (g/d); mean (±SD) | 176.58 (112.59) ^a^ | 179.48 (101.10) ^a^ | 194.91 (101.31) ^a^ | 193.86 (107.96) ^a^ | 0.211 |
|  | Legumes (g/d); mean (±SD) | 15.14 (21.95) ^a^ | 16.69 (19.10) ^a^ | 15.64 (26.50) ^a^ | 15.34 (22.40) ^a^ | 0.916 |
|  | Fruits and nuts (g/d); mean (±SD) | 225.68 (171.59) ^a^ | 217.04 (160.53) ^a^ | 211.79 (167.64) ^a^ | 239.31 (198.98) ^a^ | 0.454 |
|  | Dairy products (g/d); mean (±SD) | 265.13 (149.11) ^a^ | 309.90 (161.76) ^a,b^ | 337.44 (191.71) ^b^ | 300.31 (175.16) ^a,b^ | <0.001 |
|  | Cereals (g/d); mean (±SD) | 157.80 (77.60) ^a^ | 183.19 (89.79) ^b^ | 199.10 (85.32) ^b,c^ | 202.04 (104.89) ^b,d^ | <0.001 |
|  | Meat (g/d); mean (±SD) | 119.30 (55.96) ^a^ | 157.51 (79.34) ^b^ | 183.43 (87.79) ^c,d^ | 200.10 (97.34) ^d^ | <0.001 |
|  | Fish and seafood (g/d); mean (±SD) | 48.02 (48.63) ^a^ | 64.65 (54.01) ^b^ | 74.05 (53.25) ^b^ | 113.74 (74.65) ^c^ | <0.001 |
|  | MUFA/SFA (ratio/d); mean (±SD) | 1.98 (0.60) ^a^ | 1.89 (0.51) ^a^ | 1.86 (0.52) ^a^ | 1.90 (0.52) ^a^ | 0.207 |
|  | Alcohol (g/d); mean (±SD) | 5.26 (12.92) ^a,b^ | 9.63 (18.79) ^a,b,c^ | 12.46 (19.00) ^c^ | 14.40 (22.34) ^c^ | <0.001 |

Continuous variables were presented as means (±SD). Differences in variables across quartiles were tested using ANOVA test. Values with different superscript letter are statistically significant different (after multiple comparison post-hoc Bonferroni test). * MDA score (Trichopoulou et al., 2003).

Abbreviations. g/d: grams per day, max: maximum, MDA: Mediterranean diet adherence, min: minimum, MUFA: monounsaturated fatty acids, SD: standard deviation, SFA: saturated fatty acids.

**REFERENCES:**

Trichopoulou, A., Costacou, T., Bamia, C., Trichopoulos, D., 2003. Adherence to a mediterranean diet and survival in a Greek population. N. Engl. J. Med. 348, 2599–2608. doi:10.1056/NEJMoa025039.
